# Luxeptinib interferes with LYN-mediated activation of SYK and modulates BCR signaling in lymphoma

**DOI:** 10.1371/journal.pone.0277003

**Published:** 2023-03-08

**Authors:** Himangshu Sonowal, William G. Rice, Stephen B. Howell

**Affiliations:** 1 Moores Cancer Center, Division of Hematology, Department of Medicine, University of California, San Diego, San Diego, California, United States of America; 2 Aptose Biosciences, Inc., San Diego, California, United States of America; Hungarian Academy of Sciences, HUNGARY

## Abstract

Luxeptinib (LUX) is a novel oral kinase inhibitor that inhibits FLT3 and also interferes with signaling from the BCR and cell surface TLRs, as well as activation of the NLRP3 inflammasome. Ongoing clinical trials are testing its activity in patients with lymphoma and AML. This study sought to refine understanding of how LUX modulates the earliest steps downstream of the BCR following its activation by anti-IgM in lymphoma cells in comparison to ibrutinib (IB). LUX decreased anti-IgM-induced phosphorylation of BTK at Y551 and Y223 but its ability to reduce phosphorylation of kinases further upstream suggests that BTK is not the primary target. LUX was more effective than IB at reducing both steady state and anti-IgM-induced phosphorylation of LYN and SYK. LUX decreased phosphorylation of SYK (Y525/Y526) and BLNK (Y96) which are necessary regulators of BTK activation. Further upstream, LUX blunted the anti-IgM-induced phosphorylation of LYN (Y397) whose activation is required for phosphorylation of SYK and BLNK. These results indicate that LUX is targeting autophosphorylation of LYN or a step further upstream of LYN in the cascade of signal generated by BCR and that it does so more effectively than IB. The fact that LUX has activity at or upstream of LYN is important because LYN is an essential signaling intermediate in multiple cellular signaling processes that regulate growth, differentiation, apoptosis, immunoregulation, migration and EMT in normal and cancer cells.

## Introduction

The B-cell receptor (BCR) signaling pathway plays a key role in B-cell development [1]. Apart from its role in B-cell development, aberrant activation of BCR signaling drives proliferation of B-cell leukemias and lymphomas and is a target for therapy [2, 3]. Downstream of the BCR receptor, multiple protein kinases, linker proteins and transcription factors participate in transducing signals from the BCR. The BCR complex is a heterodimer of Igα/Igβ (CD79A/B). Following antigen-induced aggregation of the BCR, the cytoplasmic immunoreceptor tyrosine-based activation motifs (ITAM) domains of the BCR are phosphorylated by LYN, a member of the Src family kinase [4–6]. This phosphorylation-induced activation of ITAMs creates docking sites for SYK, a non-receptor tyrosine kinase, which is a necessary component of the BCR signaling pathway. LYN also phosphorylates and activates BCR co-receptor CD19 leading to activation of PI3K and generation of PIP3. PIP3 is an important downstream component of the BCR signalosome and potentiates BTK signaling by interacting with BTK-PH domains [7, 8]. Another crucial component of the BCR signalosome is the adaptor protein BLNK (also known as SLP65, BASH). SYK-mediated phosphorylation of BLNK at multiple tyrosine residues (Y72, Y84, Y96, Y178 and Y189) leads to activation of BLNK’s ability to interact with BTK [9–11]. This interaction translocates BTK to the cell membrane and increases the susceptibility of BTK to LYN- and SYK-mediated phosphorylation of BTK at Y551. Phosphorylation of BTK at Y551 leads to increased autophosphorylation at Y223 resulting in enhanced BTK activity [12]. PLCγ2 is the primary substrate of BTK and activated BTK phosphorylates and activates PLCγ2 ultimately leading to the activation of MAP kinases and transcription factors such as PKC, NF-κB, AP-1, and NFATs [3].

The BCR signalosome has a central role in regulating cellular functions in B-cells [3, 13, 14]. Ibrutinib was the first BTK inhibitor approved by FDA. It is an irreversible BTK inhibitor that covalently binds to Cys-481 in the BTK kinase domain [15]. The importance of the BCR pathway is demonstrated by the fact that IB is highly effective in the treatment of mantle cell lymphoma, chronic lymphocytic leukemia/small lymphocytic leukemia, Waldenström’s macroglobulinemia and graft-versus-host disease [16]. Inhibition of the BCR signaling pathway is also a promising therapeutic approach for ameliorating autoimmune and inflammatory complications as evidenced by recent studies [3, 16–21]. A feature of IB and other BTK inhibitors is that they regulate, to varying degrees and either directly or indirectly, the activity of multiple additional kinases such as SYK, FLT3, JAK, PI3K that affect other downstream pathways. Inhibition of these other pathways may be the basis for the activity of BTK inhibitors in non-malignant immunological disorders [22–24]. On the basis of its established ability to inhibit malignant B cells, IB was selected as the comparator as, in *in vitro* screens, LUX also inhibits recombinant wild type and C481S mutant BTK at 8.4–27 nM and 2.5–13.1 nM [25]. However, it is unclear if other kinases of the BCR signaling cascade are affected by LUX. A recent study highlighted the ability of LUX to diminish phosphorylated levels of SYK in mantle lymphoma cells [26] but the mechanisms were not clearly defined. To understand the mechanism(s) better, we analyzed the effect LUX treatment on anti-IgM-induced activation of BCR signaling in a panel of B-lymphoma cells. The results indicate that LUX is more effective than IB at reducing both steady state and anti-IgM-induced phosphorylation of LYN and SYK.

## Materials and methods

### Reagents

Luxeptinib was provided by Aptose Biosciences. Ibrutinib (#HY-10997) was purchased from MedChem Express. Goat anti-human IgM (#109-005-043) was purchased from Jackson ImmunoResearch. Human lymphoma cell lines SU-DHL-6 (#CRL-2959), JeKo-1 (#CRL-3006), RL (#CRL-2261) and Fetal Bovine Serum (#30–2020) were obtained from ATCC. SU-DHL-6 is a human lymphoblast-like cell line, JeKo-1 is a mantle cell lymphoma cell line and RL is a human non-Hodgkin’s lymphoma B cell line. RPMI-1640 medium (#11875–093) and penicillin/streptomycin (#15070063) were purchased from Thermo Fisher Scientific. Antibodies against p-BTK (Y223) (#87141), BTK (#56044), p-PLCγ2 (#3871), p-SYK (#2710), SYK (#80460), p-BLNK (#3601), BLNK (#36438), p-CD79A (#5173), p-LYN/LCK/HCK/BLK (#70926), p-LYN (Y507) (#2731), LYN (#2796, #4576), pSrc family (#6943) and GAPDH (#2118) were purchased from Cell Signaling Technology. Phospho-BTK (Y551) (#ab40770) was purchased from Abcam. PLCγ2 (#sc-5283) and CD79A (#sc-20064) were purchased from Santa Cruz Biotechnology. All other reagents and chemicals used were of analytical grade and were obtained from Sigma-Aldrich or Thermo Fisher Scientific.

### Cell culture and anti-IgM stimulation of B-cells

SU-DHL-6, JeKo-1 and RL cells were maintained in RPMI-1640 media supplemented with 10% FBS, 1X penicillin/streptomycin in a humidified incubator maintained at 5% CO_2_ and 37°C. For BCR crosslinking with anti-IgM, cells (1 x 10^6^ cells/ml) were left untreated or pretreated with the indicated concentration of LUX or IB for 2 h followed by stimulation with anti-human IgM (10 μg/ml) for 15 min. Cells were then collected by centrifugation (500 x g, 5 min, 4°C), washed with ice-cold PBS, lysed and analyzed by Western blotting.

### Co-immunoprecipitation

After the indicated treatments, the media was removed, cells were washed with ice-cold PBS and then lysed using cell lysis buffer (#9803, Cell Signaling). After a 5 min incubation in lysis buffer, the lysates were sonicated and cleared by centrifugation at 14,000 x g for 10 min at 4°C. Equal amounts of lysates were incubated with the primary antibody overnight at 4°C following manufacturer’s recommended dilution. Next day, Protein A/G agarose beads (#20421, Thermo Fisher Scientific) were added and incubated with rotation at 4°C for 4 h. The beads were pelleted by centrifugation, washed five times with cell lysis buffer and analyzed by Western blotting. Immunoprecipitation specificity was confirmed by using appropriate isotype controls.

### Western blot analysis

After the indicated treatments, cells were washed with ice-cold PBS and lysed in RIPA buffer (#sc-24948, Santa Cruz Biotechnology) or cell lysis buffer (#9803, Cell Signaling) containing protease and phosphatase inhibitors. Equal amounts of protein quantified by Bio-Rad Protein Assay Dye (#5000006) were separated by SDS-PAGE, transferred to nitrocellulose membrane and probed with the designated antibodies (1:1000 dilution). GAPDH (1:5000 dilution) was used as a loading control. The membranes were scanned using a LI-COR Odyssey IR imaging system capable of detecting antigen-antibody complexes labelled using fluorescent goat anti-rabbit (IRDye 680 RD; 1:10,000 dilution) or goat anti-mouse (IRDye 800CW; 1:10,000). Phosphoproteins were probed first, followed by stripping and re-probing the same blot with the total antibody and loading control. Quantification of bands was done using Image Studio Lite software provided with the LI-COR imaging instrument or Image J. Phosphoprotein band intensities detected by Western blotting were normalized to the levels of total protein detected by respective antibodies. All others were normalized to GAPDH. Experiments were repeated at least 3 times and a representative Western blot from one experiment is shown in the figures.

### Cytotoxicity assay

Cells were plated in 96-well plates and exposed to the indicated concentrations of drugs for 72 h. Cell viability was measured using a CCK8 kit (#CK-04, Dojindo). After 72 h exposure to drug, 10 μl CCK8 was added to the wells after which plates were incubated for 3 h in a CO_2_ incubator and then absorbance was recorded at 450 nm using a plate reader. OD450 values at 72 h were normalized to OD450 values at 0 h for each cell line. Percent viability was calculated from the DMSO control and IC_50_ values were determined using GraphPad Prism Software.

### Statistical analysis

All analysis was performed using GraphPad Prism software. Unpaired Student’s *t*-test was used for comparison between two groups. Ordinary one-way ANOVA was used for comparison between multiple groups. P < 0.05 was considered statistically significant. Quantification of Western blot data is mean ± SEM of 2 independent experiments unless otherwise stated.

## Results

### LUX downregulates phosphorylation of BTK and kinases upstream of BTK

**Fig 1:**
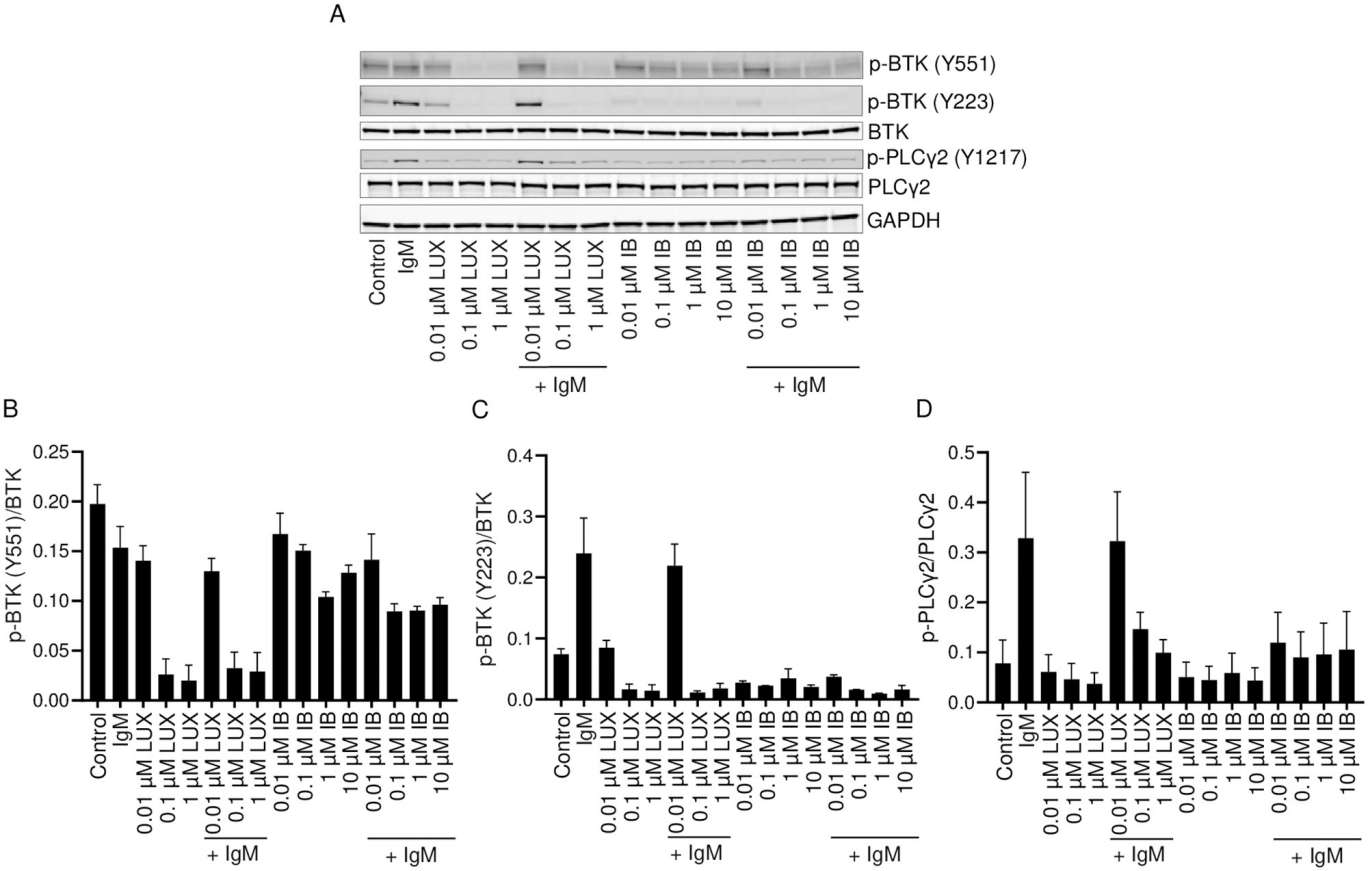
Effect of LUX and IB on anti-IgM-induced BTK activation in SU-DHL-6 cells. SU-DHL-6 cells were pre-treated for 2 h with either LUX or IB and then stimulated with anti-human IgM or left unstimulated. (A) Western blot analysis of whole cell lysates for p-BTK (Y551 and Y223), BTK, p-PLCγ2, PLCγ2 and GAPDH; representative blots are shown. (B-D) Quantification of intensity of bands in Western blots shown in A. Data is mean ± SEM from 2 independent experiments.

The ability of LUX and IB to reduce the phosphorylation of BTK at Y551 was examined in SU-DHL-6 large cell lymphoma cells with and without activation of BCR by a 15 min exposure to anti-IgM. Cells were exposed to LUX and IB at concentrations ranging from 0.1–1 μM for 2 h prior to anti-IgM treatment. Stimulation with anti-IgM alone for 15 min did not change the phosphorylation levels of BTK at Y551. However, a 2 h treatment with LUX produced a concentration-dependent decrease in the levels of phosphorylated BTK (Y551), both in the absence and in presence of anti-IgM stimulation (Fig 1A and 1B). A minimal concentration-dependent reduction in the level of phosphorylated BTK (Y551) was also observed in IB-treated anti-IgM stimulated cells but LUX was 100-fold more potent than IB. Unlike the situation for BTK Y551, a 15 min exposure to anti-IgM alone produced a marked increase in phosphorylation of BTK at Y223. In the absence of BCR activation with anti-IgM, both drugs were approximately equipotent and each produced a modest decrease in phosphorylation of BTK at Y223. However, IB was 10-fold more potent than LUX at inhibiting the anti-IgM-induced phosphorylation of BTK at Y223 (Fig 1A and 1C). Thus, both with and without BCR activation, in the SU-DHL-6 cells LUX was substantially more potent than IB at reducing phosphorylation at Y551, but IB was more potent than LUX with respect to targeting phosphorylation at Y223. Y223 is the site at which BTK phosphorylates itself after activation due to phosphorylation at Y551. These results were replicated in two other lymphoma cell lines, JeKo-1 and RL, with the exception that in the JeKo-1 cells there was some up-regulation of phosphorylation at BTK (Y551) by exposure to anti-IgM alone (S1 Fig). Thus, these results suggest that LUX is more potent at inhibiting the phosphorylation that activates BTK, but IB is more potent at blocking the ability of activated BTK to autophosphorylate. Thus, both drugs are expected to reduce downstream signaling from BTK but by distinct mechanisms.

PLCγ2 is a well-documented substrate for activated BTK. When lysates were probed for phosphorylated PLCγ2, IB was found to be 10-fold more potent than LUX at inhibiting anti-IgM-induced phosphorylation of PLCγ2 at Y1217 (Fig 1A and 1D). Thus, while LUX is much better than IB at preventing phosphorylation of BTK at Y551, this does not translate into an ability to effectively prevent BTK from autophosphorylating at Y223 and engaging a major downstream substrate.

**Fig 2:**
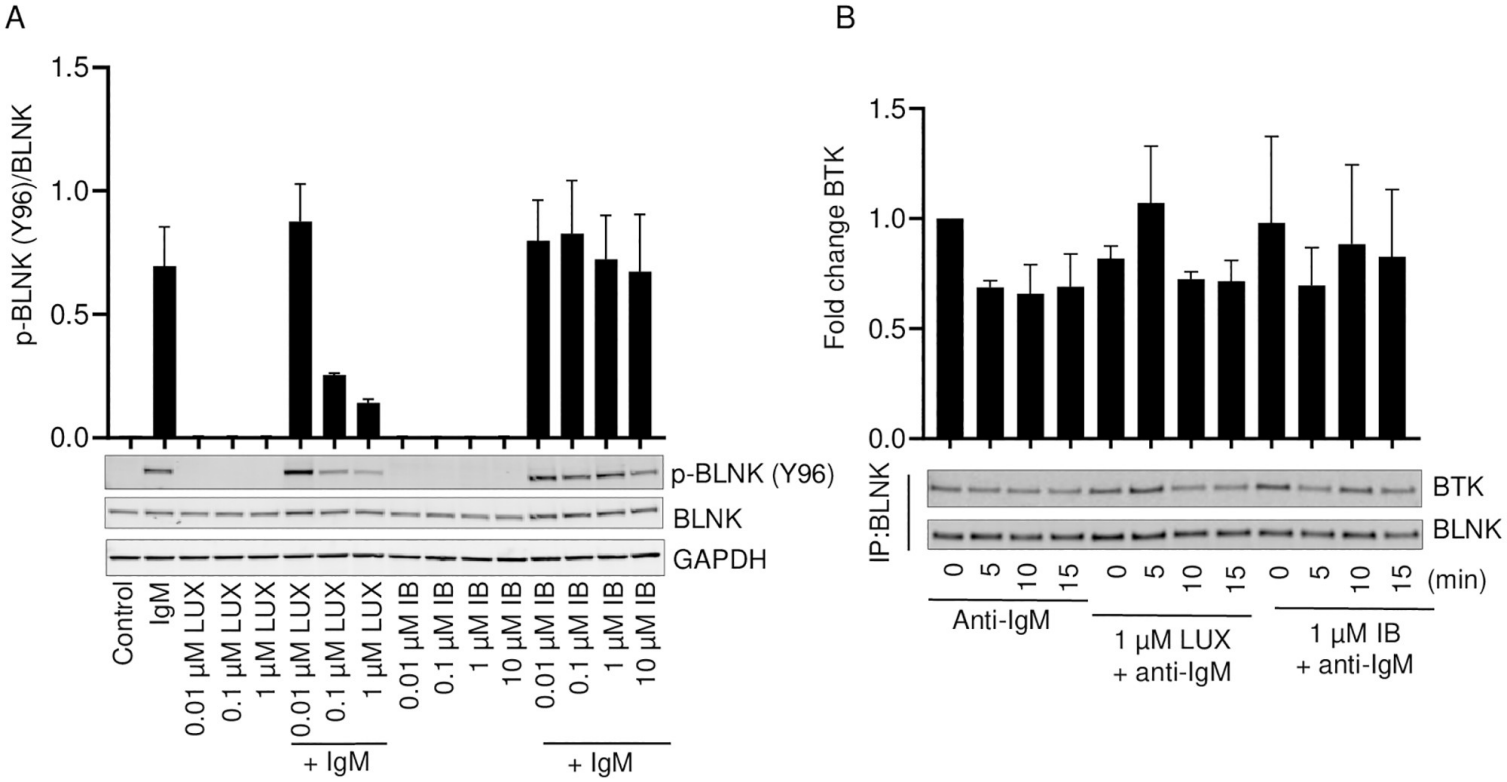
Effect of LUX and IB on anti-IgM-induced activation of BLNK and BLNK-BTK interactions in SU-DHL-6 cells. (A) Western blot analysis of whole cell lysates for p-BLNK (Y96), BLNK and GAPDH in SU-DHL-6 cells pre-treated for 2 h with either LUX or IB and then stimulated with anti-human IgM or left unstimulated. (B) Representative blots showing BLNK immunoprecipitates probed for BTK and BLNK in SU-DHL-6 cells sampled at 5, 10 and 15 min post anti-IgM stimulation. Data is mean ± SEM of data from 2 independent experiments.

BLNK is an adaptor protein that is phosphorylated in response to BCR stimulation and plays an important role in BTK activation. SYK-mediated phosphorylation of five canonical YXXP tyrosine residues (Y72, Y84, Y96, Y178 and Y189) in the acidic and proline-rich domains of BLNK induces its association with SH2 domain-containing proteins such as Vav, BTK and PLCγ2 with the phosphorylated tyrosines on BLNK leading to their activation [10, 27, 28]. Thus, BLNK is of interest with respect to the question of how LUX reduces the level of BTK phosphorylated at Y551. LUX inhibited anti-IgM-induced phosphorylation of BLNK (Y96) in a concentration dependent manner in SU-DHL-6 whereas IB had no effect on phospho-BLNK (Y96) (Fig 2A). This provides further evidence of a difference in kinase targets between LUX and IB, and suggests that the most important target of LUX lies between BCR and BLNK in the cascade. Since LUX reduced the levels of BLNK that is phosphorylated following anti-IgM- stimulation, and BLNK is an adaptor protein that has binding sites for BTK and PLCγ2, we sought to determine whether LUX interferes with binding of BTK to BLNK. Immunoprecipitation of BLNK from SU-DHL-6, JeKo-1 and RL cell lysates and probing with anti-BTK indicated that neither LUX nor IB significantly affected binding of BTK to BLNK (Fig 2B, S2 Fig).

**Fig 3:**
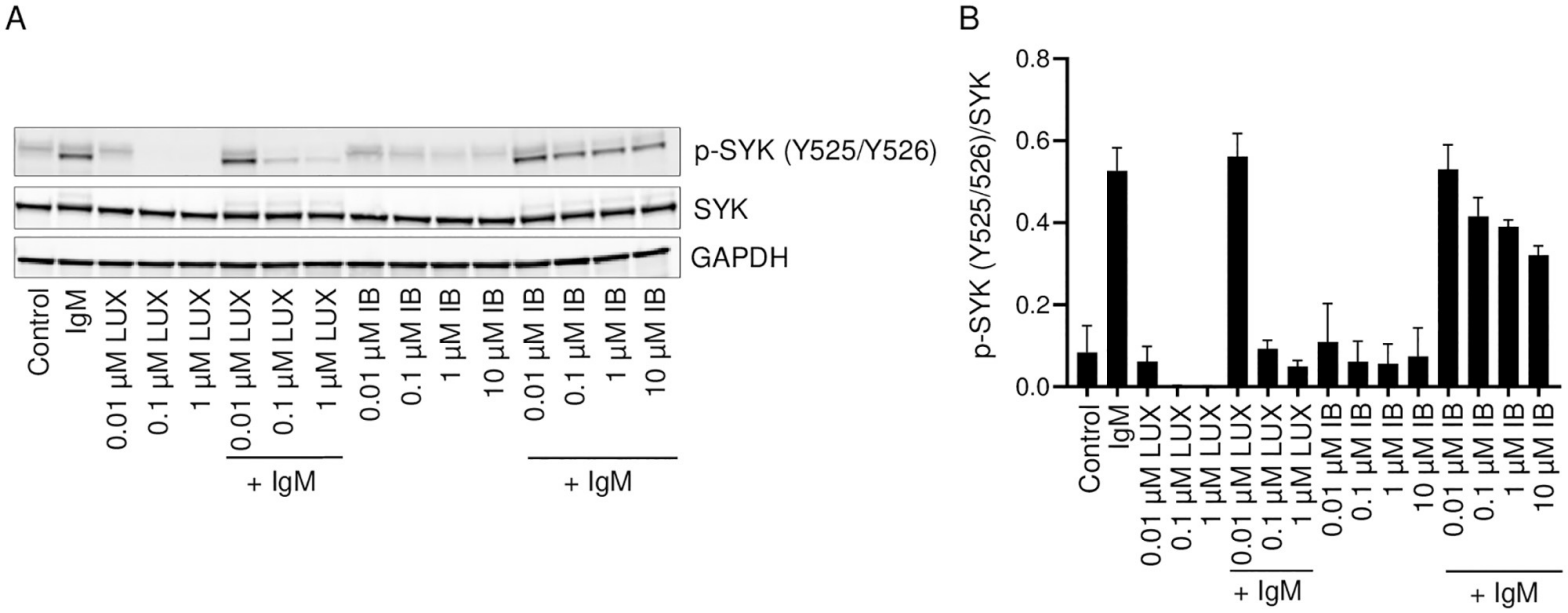
Effect of LUX and IB on anti-IgM-induced activation of SYK in SU-DHL-6 cells. SU-DHL-6 cells were pre-treated for 2 h with either LUX or IB and then stimulated with anti-human IgM or left unstimulated. (A) Western blot analysis of whole cell lysates for p-SYK (Y525/Y526), SYK, and GAPDH; representative blots are shown. (B) Quantification of intensity of bands in Western blots shown in A. Data is mean ± SEM of data from 2 independent experiments.

Further upstream of BLNK in the BCR signaling cascade, spleen tyrosine kinase (SYK) is another important component of the BCR signaling pathway that is phosphorylated after stimulation of the BCR with anti-IgM. A 2 h treatment with LUX produced a concentration-dependent decrease in the levels of phosphorylated SYK (Y525/Y526) in both the absence and presence of anti-IgM stimulation in SU-DHL-6 cells (Fig 3A and 3B). The ability of LUX to reduce steady-state and anti-IgM-induced phosphorylation of SYK was more than 100-fold greater for LUX than for IB and was observed in all the three lymphoma cell lines studied (Fig 3, S1 Fig). Since SYK does not autophosphorylate, this result indicates that an important target of LUX, but not IB, lies upstream of SYK in the BCR cascade.

### Effect of LUX and IB on LYN

**Fig 4:**
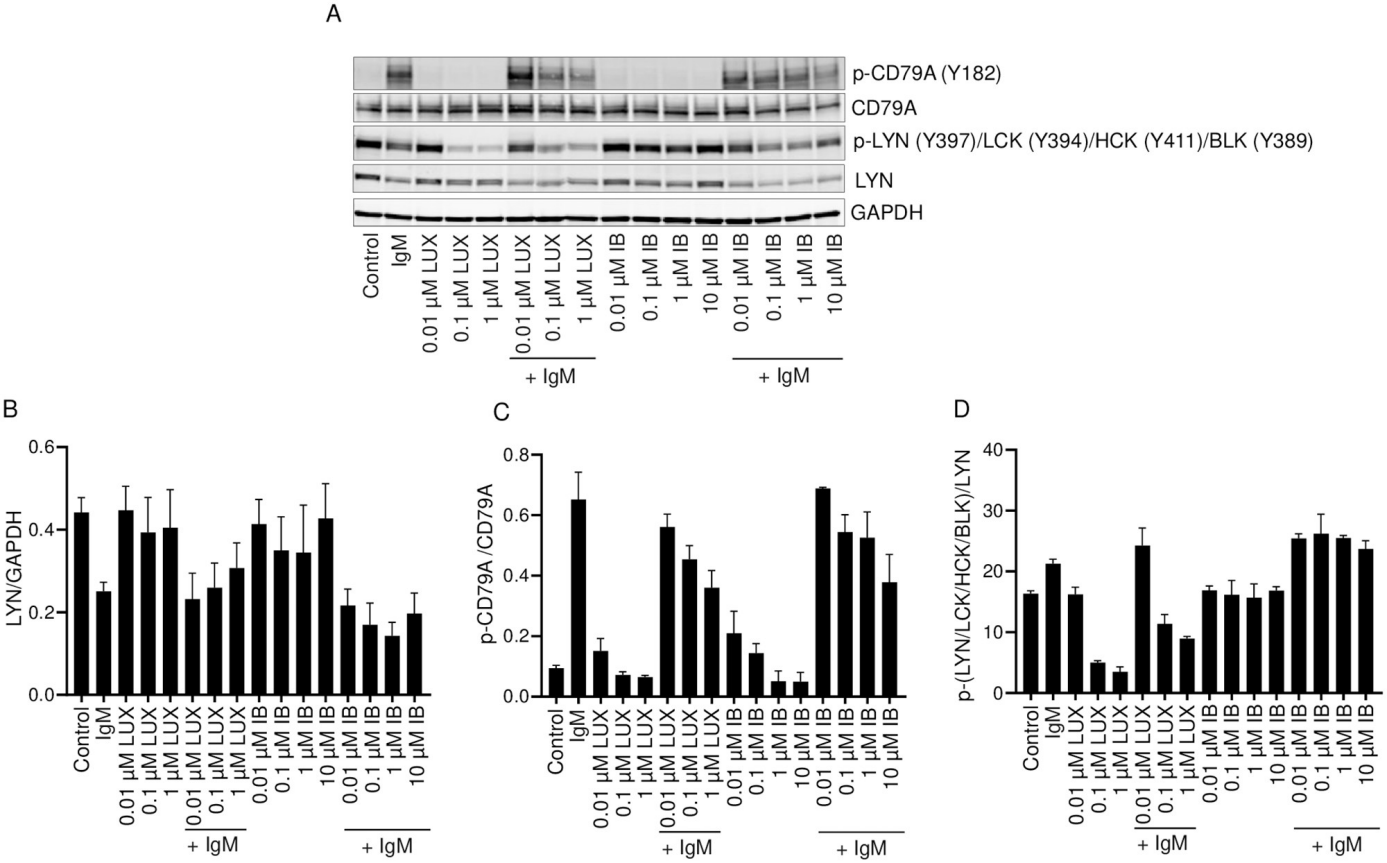
Effect of LUX and IB on LYN and CD79A in SU-DHL-6 cells. SU-DHL-6 cells were pre-treated for 2 h with either LUX or IB and then stimulated with anti-human IgM or left unstimulated. (A) Western blot analysis of whole cell lysates for p-CD79A (Y182), CD79A, p-LYN (Y397)/LCK (Y394)/HCK (Y411)/BLK (Y389), LYN and GAPDH; representative blots are shown. (B-D) Quantification of intensity of bands in Western blots shown in A. Data is mean ± SEM of data from 2 independent experiments.

LYN is a member of the Src kinase family that is a key upstream regulator of the BCR signaling pathway. Following activation, proteasome-mediated degradation of LYN is a necessary event in the regulation of BCR signaling [29]. We observed that the total levels of LYN were significantly lower in anti-IgM stimulated cells (Fig 4A and 4B) consistent with activation-induced degradation. A 2 h treatment with LUX or IB alone had no effect on the total levels of LYN in the absence of anti-IgM stimulation. IB had no effect on anti-IgM-induced decrease in total LYN; however, LUX blocked this effect of anti-IgM even at a concentration of 0.01 μM. Since LUX reduced the phosphorylation of LYN but IB did not, this suggests that BCR activation reduces LYN levels by a mechanism that is blocked by LUX.

Upon antigen stimulation of BCR, an activated form of LYN phosphorylates the cytoplasmic immunoreceptor tyrosine-based activation motifs (ITAMs) of the immunoglobulin α (Ig-α) and Ig-β domains (CD79A/CD79B) of BCR leading to its activation and the activation of other downstream components such as SYK. When the lysates were probed for phospho-CD79A, it was found that the anti-IgM-induced phosphorylation of CD79A (Y182) was inhibited by LUX in a concentration-dependent manner, whereas IB had no effect (Fig 4A and 4C). This provides further evidence of a differential effect of the two drugs on the activity of LYN.

The activation of LYN involves phosphorylation on Y397 and dephosphorylation of the inhibitory Y507 site [30, 31]. We first determined the effect of LUX using an antibody which, apart from detecting phosphorylated LYN (Y397), also detects phosphorylated LCK (Y394), HCK (Y411) and BLK (Y389). As shown in Fig 4A and 4D, stimulation with anti-IgM increased the level of phosphorylation detected by this antibody on LYN (Y397) as well as that on the other kinases but there was no clear effect when the cells were exposed to IB alone or when treated first with IB and then stimulated with anti-IgM except at a concentration of 10 μM which is a level that cannot be attained in the plasma of patients receiving this drug. In contrast, LUX produced a concentration-dependent decrease in the level of phosphorylation, both in the absence and presence of anti-IgM stimulation at concentrations of 0.1 and 1 μM. Steady-state plasma levels of LUX in the range of 1 μM were observed during BID dosing in mice [32] and data from recent clinical trials indicate that LUX plasma levels of 1 μM or greater are attained in patients with AML, CLL and lymphomas [33, 34]. The differences in the behavior of LUX and IB with respect to reduction in phosphorylation of LYN and CD79A, including the effect of both the drugs on anti-IgM-induced reduction in total levels of LYN, was also observed in both the JeKo-1 and RL cells as shown in S1 Fig.

**Fig 5:**
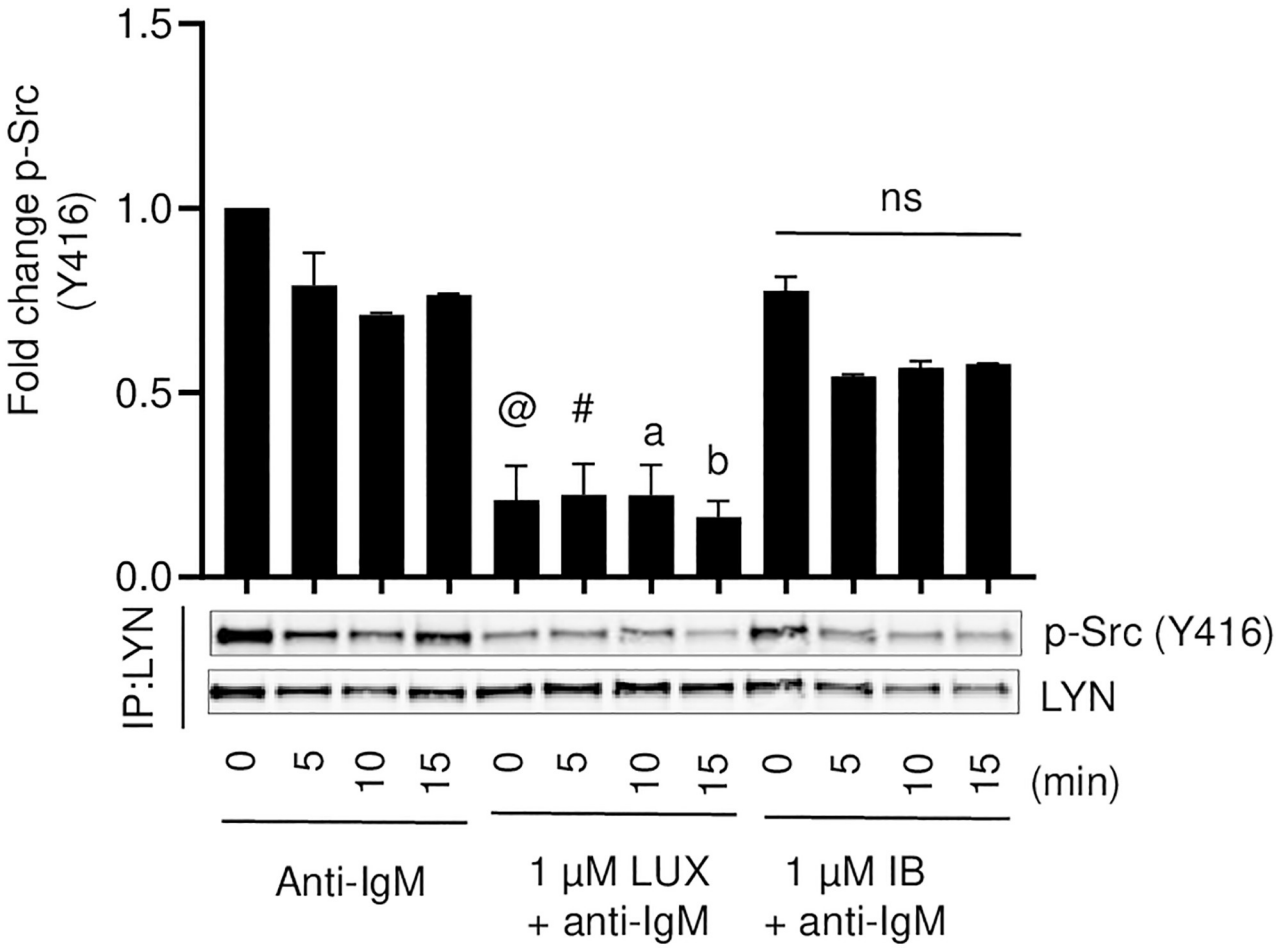
Effect of LUX and IB on anti-IgM-induced phosphorylation of LYN in SU-DHL-6 cells. Cells were pre-treated with vehicle or 1 μM LUX or IB after which they were stimulated with anti-human IgM. LYN immunoprecipitates from these cells were analyzed using antibodies for phospho-Src (Y416) and LYN at the indicated time points in SU-DHL-6 cells. Histograms show quantification of phospho-Src (Y416) band intensity relative to that of LYN. Data is mean ± SEM of data from 2 independent experiments. In the LUX + anti-IgM stimulated group, a statistically significant inhibition of p-Src (Y416) is denoted by @ (p<0.0001), # (p = 0.0003), a (p = 0.001), b (p = 0001) when compared to the respective time point for the anti-IgM stimulated cells. No significant inhibition of p-Src (Y416) was observed in the IB + anti-IgM group when compared to the respective time point for the anti-IgM stimulated cells.

To specifically determine the effect on phosphorylation of LYN (Y397), the effect of LUX and IB on phosphorylation of LYN (Y397) was further confirmed by immunoprecipitating LYN and probing the LYN immunoprecipitates with an antibody for phospho-Src family kinase (Y416). LYN (Y397) is located in the catalytic domain and is structurally equivalent to Y416 of the Src family kinases. Phosphorylation at Y397 correlates with enzyme activation of LYN [30, 31]. Probing for phospho-Src (Y416) in LYN immunoprecipitates thus provides an indirect measure of LYN activation. Data presented in Fig 5 (SU-DHL-6 cells) and S3 Fig (JeKo-1 and RL cells) indicate that the phosphorylation of Y397 on LYN that was detected by phospho-Src (Y416) antibody was reduced by LUX but not by IB which had no effect at a concentration of 1 μM. Thus, a clear differential effect of LUX and IB was detected by both approaches to detecting the phosphorylation of LYN.

**Fig 6:**
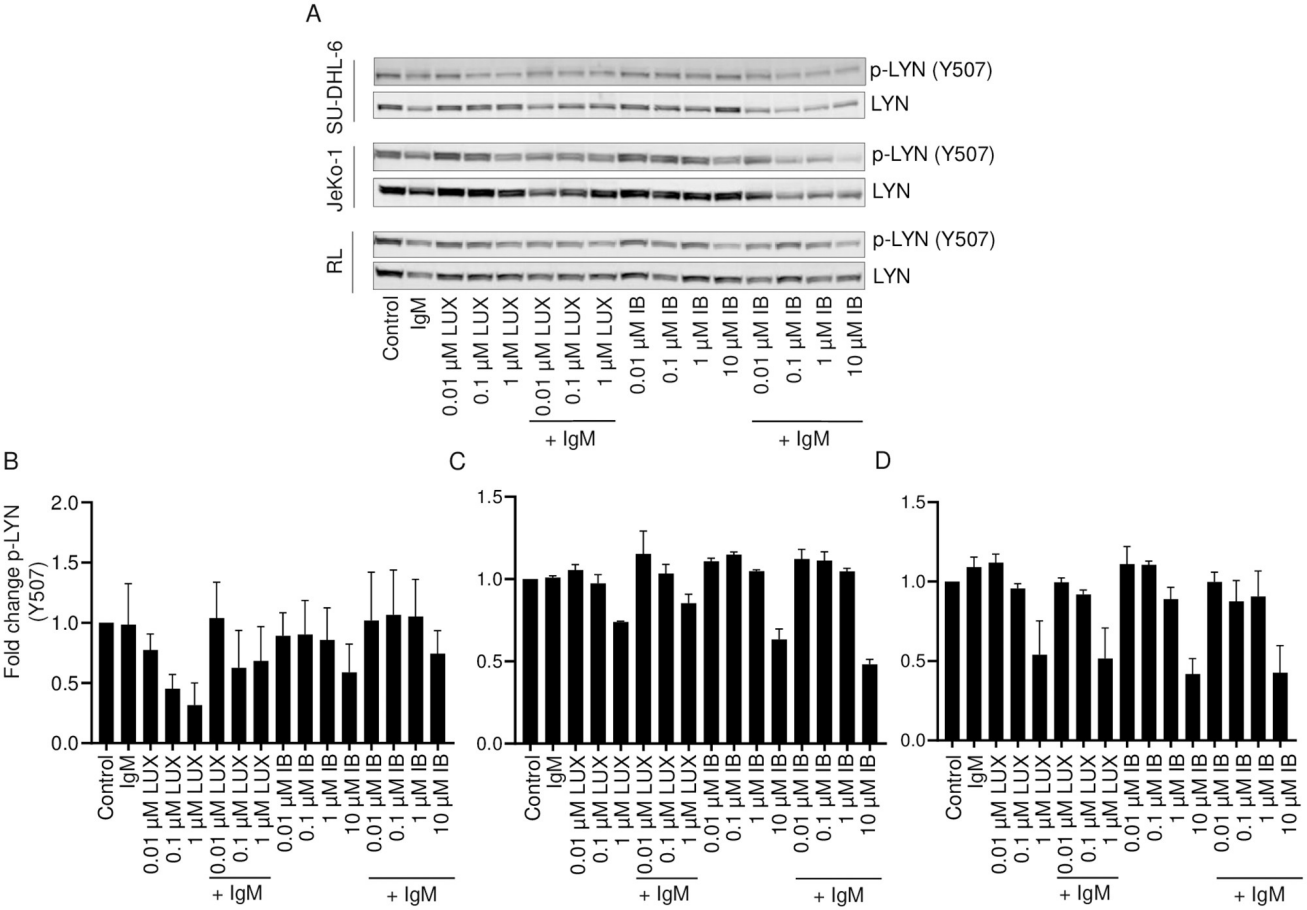
Effect of LUX and IB on IgM-induced phosphorylation of LYN (Y507) in SU-DHL-6, JeKo-1 and RL cells. Cells were pre-treated for 2 h with either LUX or IB following which they were stimulated with anti-human IgM or left unstimulated. (A) Whole cell lysates were analyzed by Western blotting for phospho-LYN (Y507) and LYN; representative blots are shown. (B-D) Quantification of intensity of bands in Western blots shown in A. Data is mean ± SEM of data from 2 independent experiments.

Dephosphorylation of the inhibitory tyrosine Y507 in the C-terminal domain of LYN is a necessary event for LYN activation. When lysates were probed for phospho LYN (Y507), no effect of either LUX or IB in the absence or presence of anti-IgM stimulation was observed in SU-DHL-6 cells, JeKo-1 or RL cells (Fig 6A–6D). This suggests that inhibition of LYN activity by LUX is not mediated through prevention of Y507 de-phosphorylation.

## Discussion

Although both LUX and IB are potent inhibitors of recombinant forms of BTK, and of cells engineered to express wild type and some mutant forms of this enzyme, they have different efficacy profiles as observed in studies of LUX in xenograft models and in initial clinical trials in patients with CLL and lymphomas [25, 35]. In this study, we sought to discern differences in their effect on BCR signaling and identify the kinases that are most differentially affected in B-cell lymphoma cells. The results indicate that the two drugs have quite different effects on the phosphorylation of BTK at Y223 and Y551, and on BTK activity, but that LUX affects the phosphorylation of multiple kinases and adaptor proteins that lie upstream of BTK in the BCR activation pathway under circumstances where IB has little or no effect.

Following BCR activation, BTK is phosphorylated at Y551 and Y223. Y551 is located in the activation loop and can be either trans-autophosphorylated or phosphorylated by some upstream kinases. Y223 is located in the ligand binding pocket and regulates BTK kinase activity [36, 37]. Phosphorylation of BTK at Y551 is reported to be a necessary first event that renders BTK capable of autophosphorylation of Y223. In the lymphoma cells, IB was confirmed to be a very potent inhibitor of phosphorylation at Y223. However, despite the fact that LUX was very effective at reducing phosphorylation of BTK at Y551, it had a lesser effect on phosphorylation at Y223 and the ability of LUX to impair the activity of BTK, as detected by phosphorylation of PLCγ2 correlated with the degree of inhibition of phosphorylation of Y223. Thus, in BCR-activated lymphoma cells, phosphorylation of BTK at Y551 is not a prerequisite for autophosphorylation at Y223, or else Y223 is being phosphorylated by some other kinase.

Among the kinases upstream of BTK, SYK and members of the Src family kinase such as LYN and the adaptor protein BLNK, play important roles in the BCR signalosome [10, 38]. Phosphorylation of Y525/Y526 located in the activation loop of the SYK kinase domain is necessary for SYK activation and kinase activity [39]. SYK is of particular interest as a therapeutic target in leukemia and lymphoma as it plays a central role in activation of downstream kinases both via the BTK pathway and through its ability to directly phosphorylate and activate PI3K resulting in formation of second messenger PIP3 from PIP2 [40, 41]. In lymphoma cells we found that LUX was a very potent at reducing the phosphorylation of SYK at Y525/Y526 whereas IB was not. This identifies a second clear difference in the pharmacodynamic effects of these two drugs and provides an explanation for how LUX can affect downstream signaling in the absence of an ability to effectively inhibit phosphorylation of Y223 of BTK and BTK activity.

When activated, SYK interacts with and phosphorylates BLNK which has binding sites for BTK and PLCγ2. The association of BLNK with BTK and PLCγ2 brings both BTK and PLCγ2 into proximity and this further potentiates BTK signaling. A 5-fold increase in BTK activity was reported when BTK and PLCγ2 are bound to BLNK [9, 10, 28]. This interaction also increases the ability of SYK to directly trans-phosphorylate BTK at Y551 [10, 11]. Furthermore, BLNK is reported to be necessary for Rac1-mediated JNK activation in B-cells, a necessary component of BCR signaling [42]. Our data suggest that, although LUX reduced the levels of phosphorylated BLNK following anti-IgM stimulation, the interaction between BTK and BLNK was not altered. The ability of SYK to activate PI3K and generate PIP3 from PIP2 is mediated by the BCR co-receptor CD19 and other associated proteins such as BCAP (B-cell adaptor for PI3K) and GAB1/2 (GRB2-associated protein 1 and 2). All these processes enhance BTK activity leading to activation of PLCγ, PKCβ, Ca^+2^ flux and other downstream effectors such as ERKs, JNK, NF-κB etc. leading to full effect of BCR signaling [43, 44]. Thus, the effects of inhibiting phosphorylation of SYK are complex. In broad kinase screens LUX was observed to be a poor direct inhibitor of SYK enzymatic activity with an IC_50_ of 59 nM (Carna Biosciences) and Kd of 180 nM (Discover X) against recombinant SYK [25].

The kinase screens also highlighted the ability of LUX to target other members of Src family kinase [25]. The Src family kinases are an integral component of BCR signalosome. The BCR complex lacks intrinsic tyrosine-kinase activity and interaction with members of the Src family (including SYK and TEC family depending on cell type) propagate signals from the BCR. In B-cells, after BCR engagement with antigen, members of the Src family kinase (LYN, FYN and BLK) are amongst the first kinases to be activated. In B-cells LYN is of particular interest because it physically interacts with the ITAMs of the BCR receptor following antigen binding [45]. Although the mechanisms are poorly defined, existing data suggest that LYN is the primary initiator kinase in the BCR signaling cascade in B-cells [45, 46].

A necessary step in the activation of LYN, as well as other members of the Src kinase family, is the dephosphorylation of inhibitory tyrosine residue Y507 in the C-terminal domain which is carried out by protein tyrosine phosphatase CD45. This induces unfolding and exposure of Y397 (equivalent to Y416 of Src) within the activation loop of LYN leading to transphosphorylation of Y397 and full activation of LYN [47, 48]. LYN is also known to undergo autophosphorylation which regulates its kinase activity [49, 50]. Western blot analysis of cell lysates using an antibody that detects phosphorylated LYN (Y397), but which also detects phosphorylated LCK (Y394), HCK (Y411) and BLK (Y389), disclosed that LUX reduced the phosphorylation signal even at a concentration of 0.1 μM whereas IB had no effect even at 10 μM. Application of an alternative approach in which phosphorylation at Src Y416 was used as a surrogate of phosphorylation of LYN (Y397) confirmed the differential effect of the two drugs. Probing with an antibody for Y507 did not disclose any effect of either LUX or IB in the absence or presence of anti-IgM stimulation raising the question of whether Y397 is phosphorylated by a different kinase rather than just through autophosphorylation when phosphorylation on Y507 is removed [51]. Studies have suggested that, independent of dephosphorylation of the inhibitory Y507 residues, members of the Src family kinases can also be activated by SH3-domain displacement [52], which could explain the activation of LYN in the absence of dephosphorylation of Y507.

Evidence that the effect of LUX on the phosphorylation of LYN resulted in reduced activity of the enzyme was provided by the observation that anti-IgM-induced phosphorylation of CD79A (Y182) was inhibited by LUX in a concentration-dependent manner. The fact that IB had no effect provided further evidence of a differential effect of the two drugs on the activity of LYN. Activation of BCR was associated with reduction in the level of total LYN consistent with anti-IgM-induced degradation as previously reported [29]. Interestingly, neither drug affected total LYN level in the absence of BCR activation, and neither drug significantly blocked the reduction after activation. Thus, whatever mechanism mediates degradation is not affected by either drug.

The question of whether the unique differences in the effect of LUX and IB on phosphorylation of kinases in the BCR pathway detectable with a 2 h exposure to drug are linked to differences in cytotoxic potency is of interest. When exposed for 72 h, LUX was found to be between 18- and 1,272-fold more potent at inhibiting growth than IB across the 3 cell lines (S1 Table) suggesting that the profile of kinase inhibition produced by LUX is more lethal than that produced by IB. However, substantial caution is required; heterogeneity in drug uptake and many other factors render confidence in such a conclusion low.

**Fig 7:**
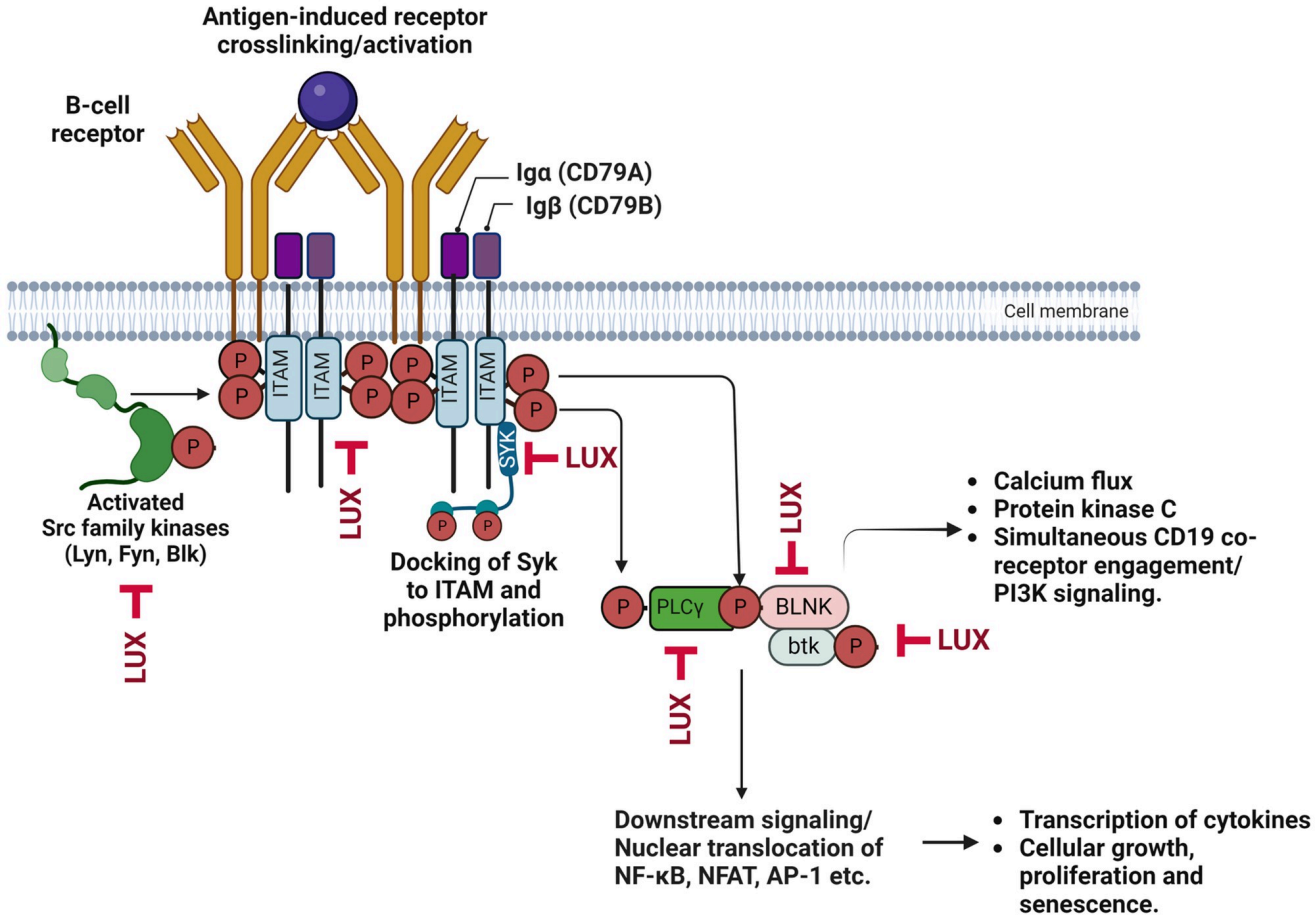
Summary of important kinases of the BCR signaling pathway whose phosphorylation is reduced by LUX following activation by anti-IgM in lymphoma cells. The figure was created with Biorender.com.

In summary, in human lymphoma cells LUX and IB have quite distinct mechanistic effects on the phosphorylation of BTK and its activity, and on the upstream kinases SYK and LYN, and the adaptor protein BLNK against which LUX is very potent and IB had little effect. The BCR pathway kinases whose phosphorylation is modulated by LUX are highlighted in Fig 7. While LYN is a strong candidate as a primary LUX target that sits at the top of the BCR cascade, the lack of specific antibodies to the phospho- forms of this protein leave some uncertainty. Moreover, the rapid proteasomal degradation of LYN following BCR aggregation poses a challenge when estimating the levels of phosphoprotein. The Src family kinases, and particularly LYN, play important roles in several autoimmune and inflammatory complications [53, 54]. The results reported here are important because, in combination with evidence that LUX inhibits TLR signaling and activation of the NLRP3 inflammasome at concentrations well below those attained in patients, they indicate that LUX as a unique kinase profile quite different from that of IB and suggest that LUX may have activity against autoimmune and inflammatory diseases of multiple types in addition to lymphomas.

## Supporting information

S1 File (PDF)

S1 Fig Effect of LUX and IB on anti-IgM-induced activation of BCR signaling in JeKo-1 and RL cells. Cells were pre-treated for 2 h with either LUX or IB and then stimulated with anti-human IgM or left unstimulated. Whole cell lysates were analyzed by Western blotting for the indicated phosphoproteins in (A) JeKo-1 and (B) RL cells. Representative blots are shown.
(TIF)

S2 Fig Effect of LUX on BLNK-BTK interactions in JeKo-1 and RL cells. Cells were left untreated or pre-treated with 1 μM LUX or IB for 2 h followed by stimulation with anti-IgM. Representative blots showing BLNK immunoprecipitates probed for BTK and BLNK in (A) JeKo-1 and (B) RL cells sampled at 5 min. Histograms show quantification of co-precipitated protein relative to that of the protein targeted by the precipitating antibody. Bars are mean ± SEM of data from 2 independent experiments.
(TIF)

S3 Fig Effect of LUX and IB on anti-IgM-induced phosphorylation of LYN in JeKo-1 and RL cells. Cells were pre-treated with vehicle or 1 μM LUX or IB for 2 h followed by stimulation with anti-human IgM for 5 min. Western blot analysis of LYN immunoprecipitates using antibodies for phospho-Src (Y416) and LYN in (A) JeKo-1 and (B) RL cells. Histograms show quantification of phospho-Src (Y416) band intensity relative to that LYN. Data is mean ± SEM of data from 2 independent experiments. Representative blots are shown. **p<0.001.
(TIF)

S1 Table Relative sensitivity of lymphoma cell lines to LUX and IB. Table showing IC_50_ values for LUX and IB in SU-DHL-6, JeKo-1 and RL cells. Data is mean ± SEM of 3-independent experiments.
(DOCX)
