# Supplementary material for: Luxeptinib interferes with LYN-mediated activation of SYK and modulates BCR signaling in lymphoma
Source: PLoS One. 2023 Mar 8;18(3):e0277003. doi: 10.1371/journal.pone.0277003 (PMC9994718; doi:10.1371/journal.pone.0277003)

Full length blots

Manuscript title: Luxeptinib interferes with LYN-mediated activation of SYK and modulates BCR signaling in lymphoma

Authors: Himangshu Sonowal[1], William G. Rice[2], Stephen B. Howell[1] *

1 *Moores Cancer Center, Division of Hematology, Department of Medicine, University of California, San Diego, CA, USA*
2 *Aptose Biosciences, Inc., San Diego, CA, USA*

Figure 1A

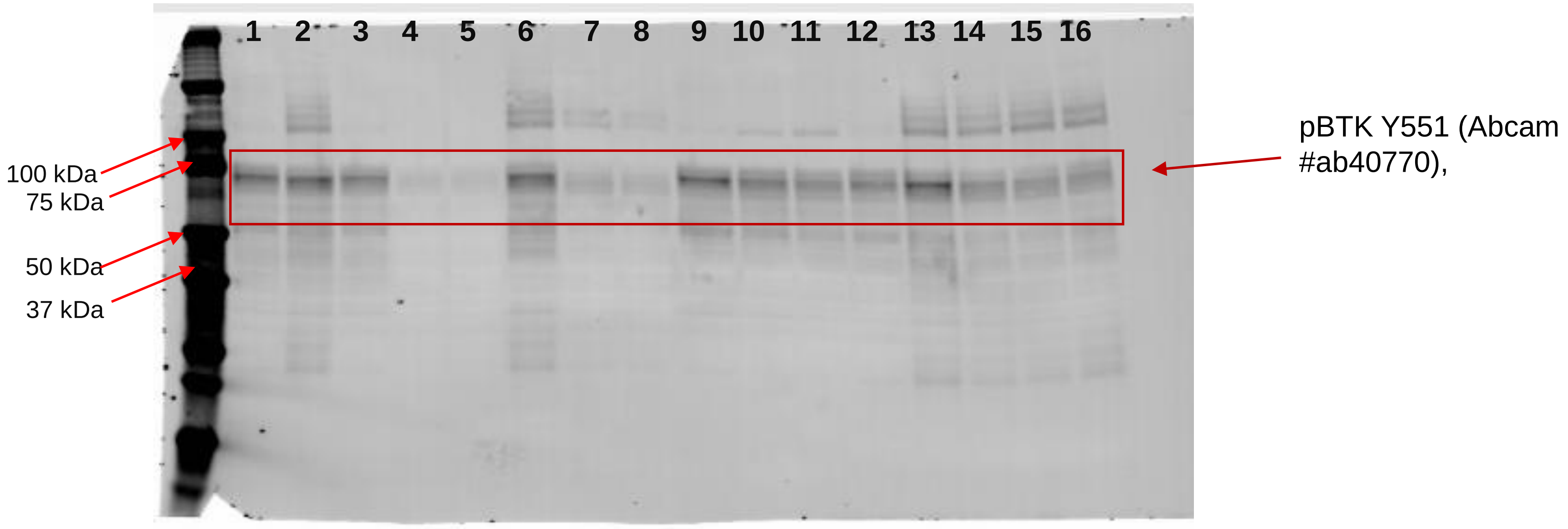

Figure 1A

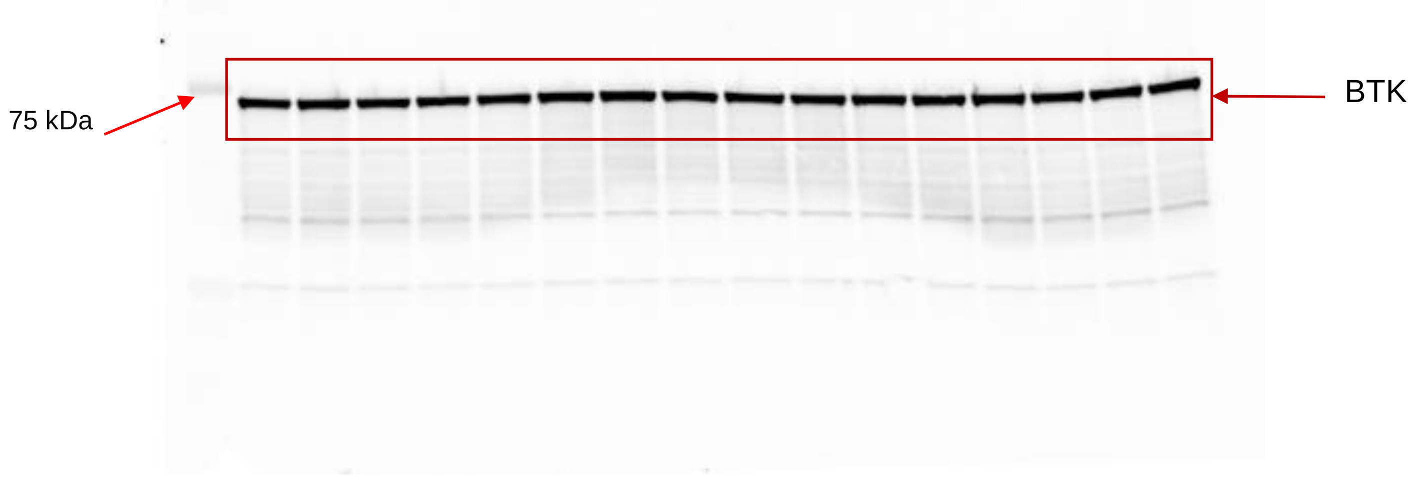

Figure 1A

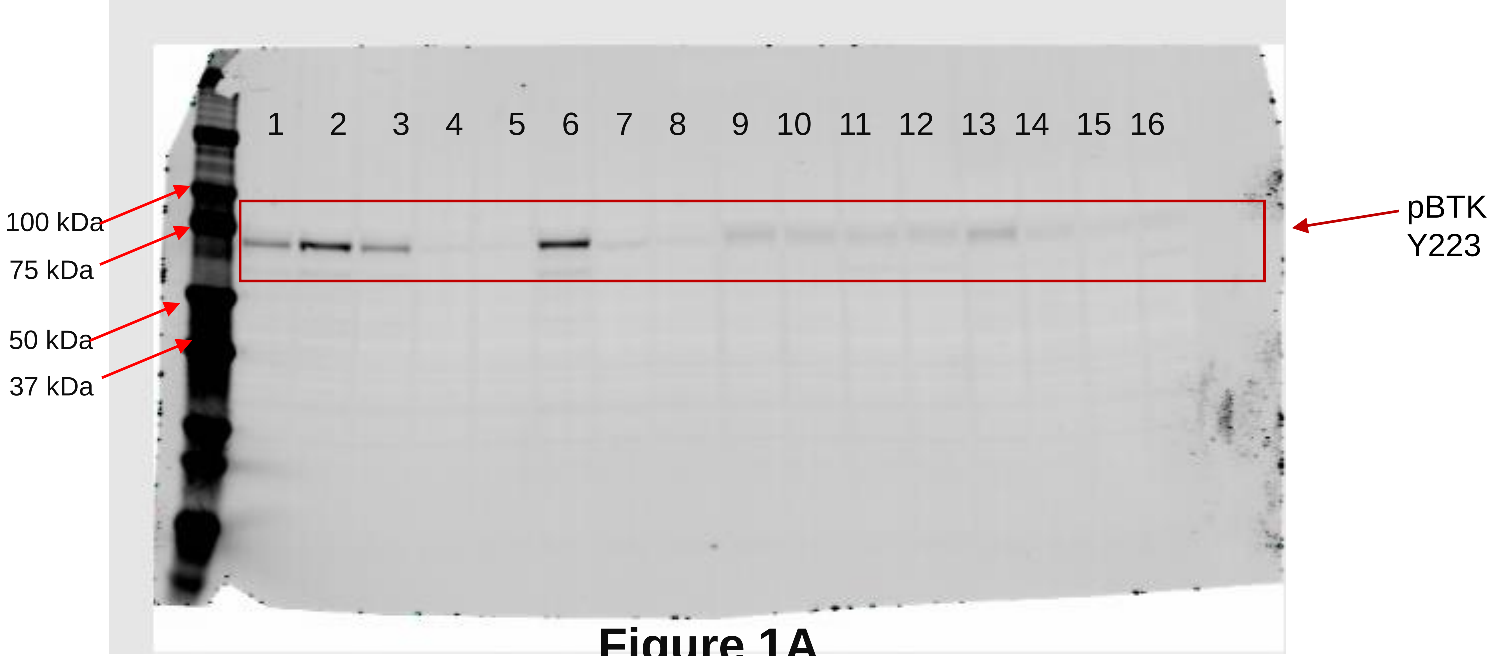

Figure 1A

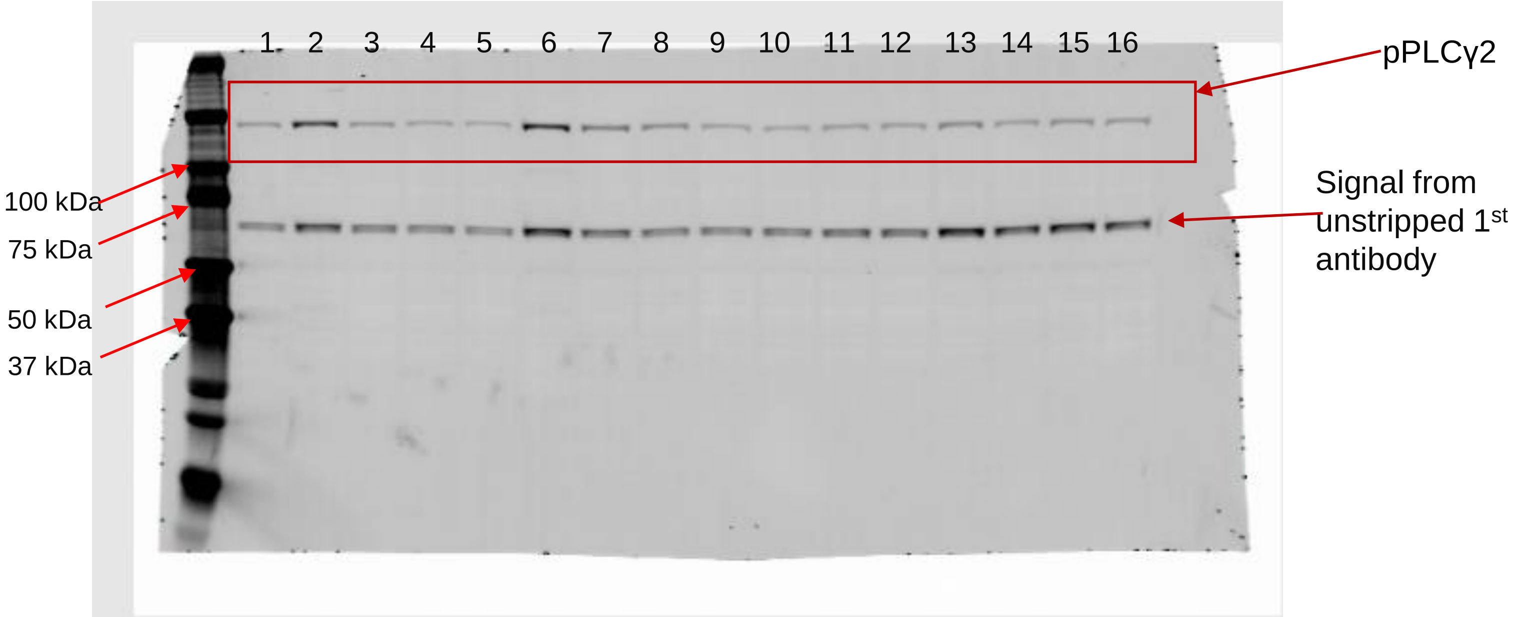

Figure 1A

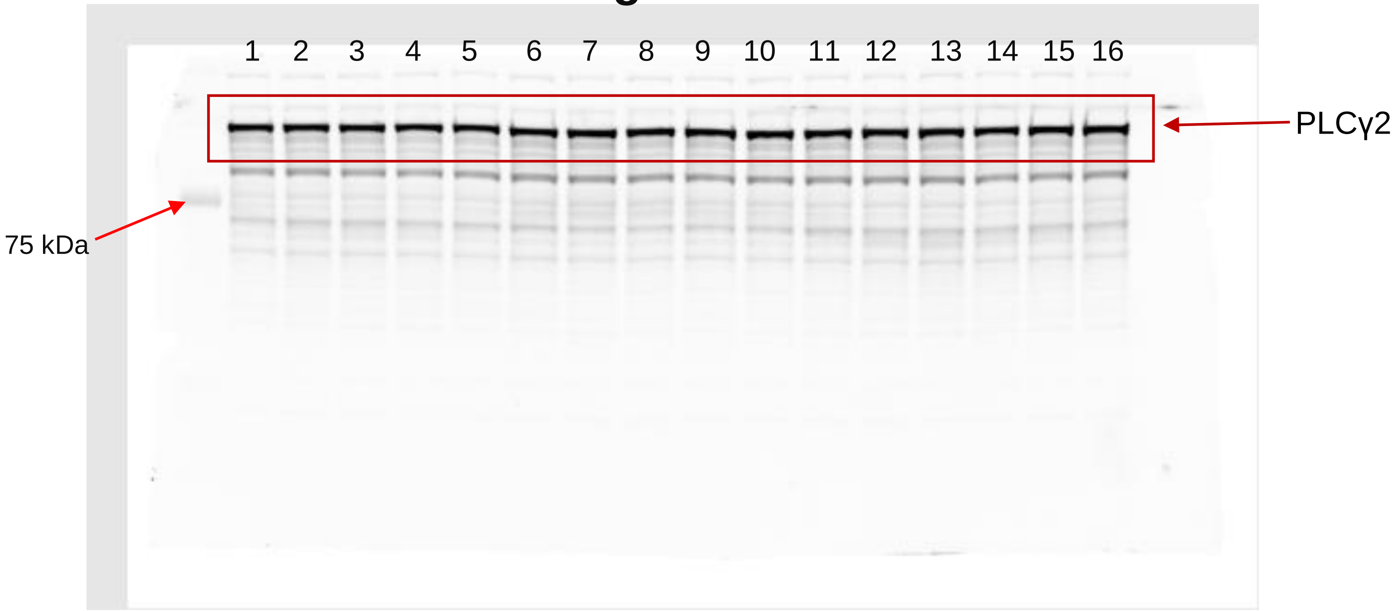

Figure 1A

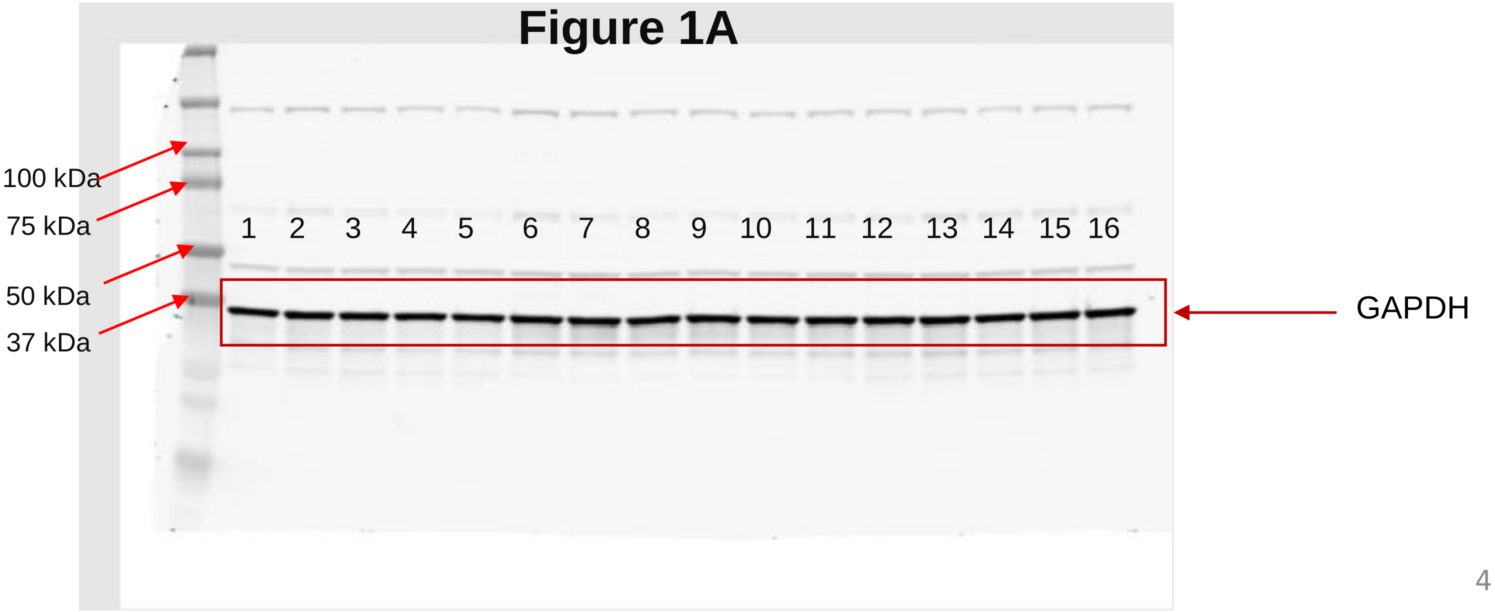

Figure 2A

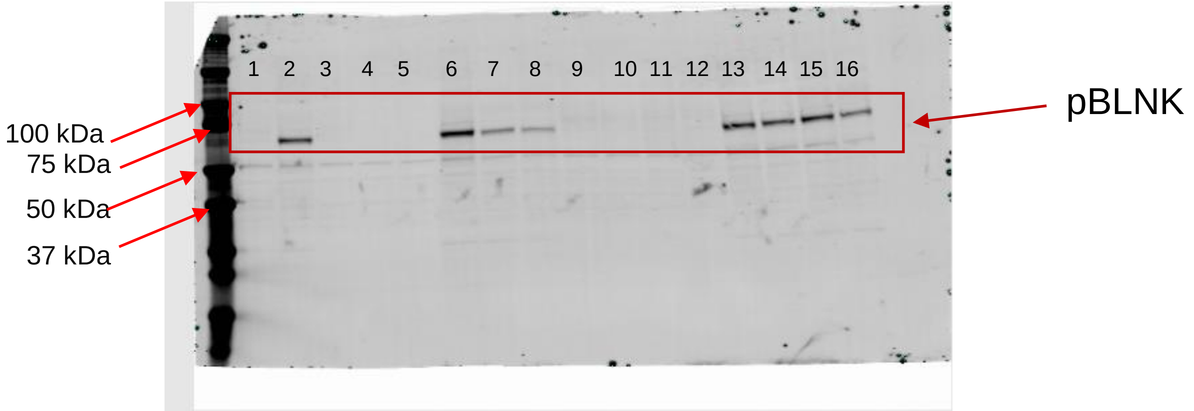

Figure 2A

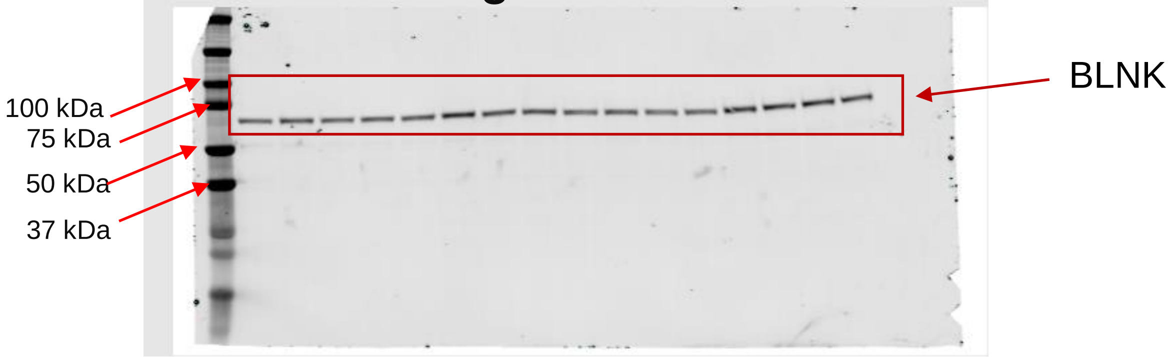

Figure 2A

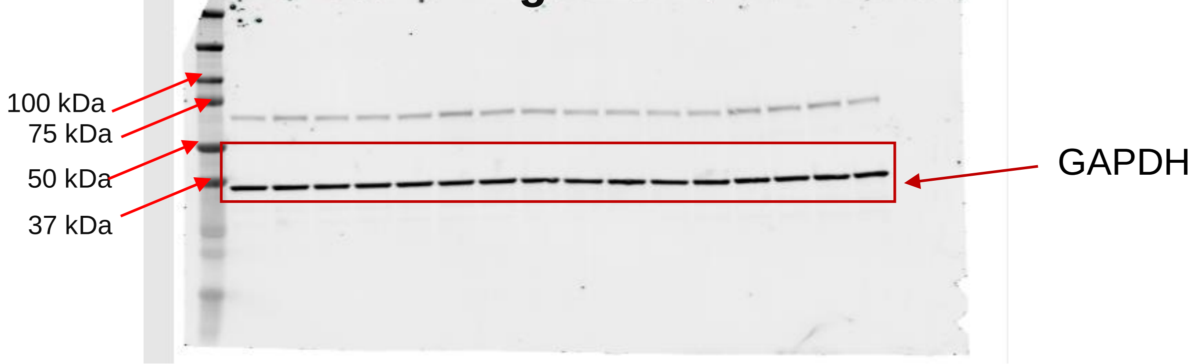

Figure 2B

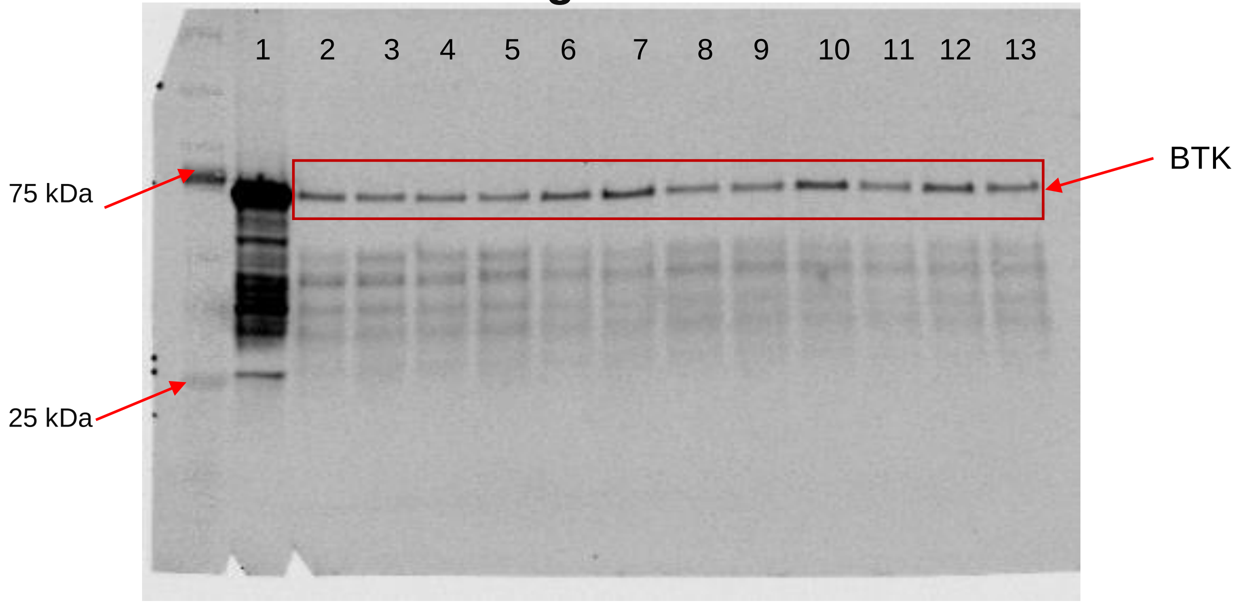

Figure 2B

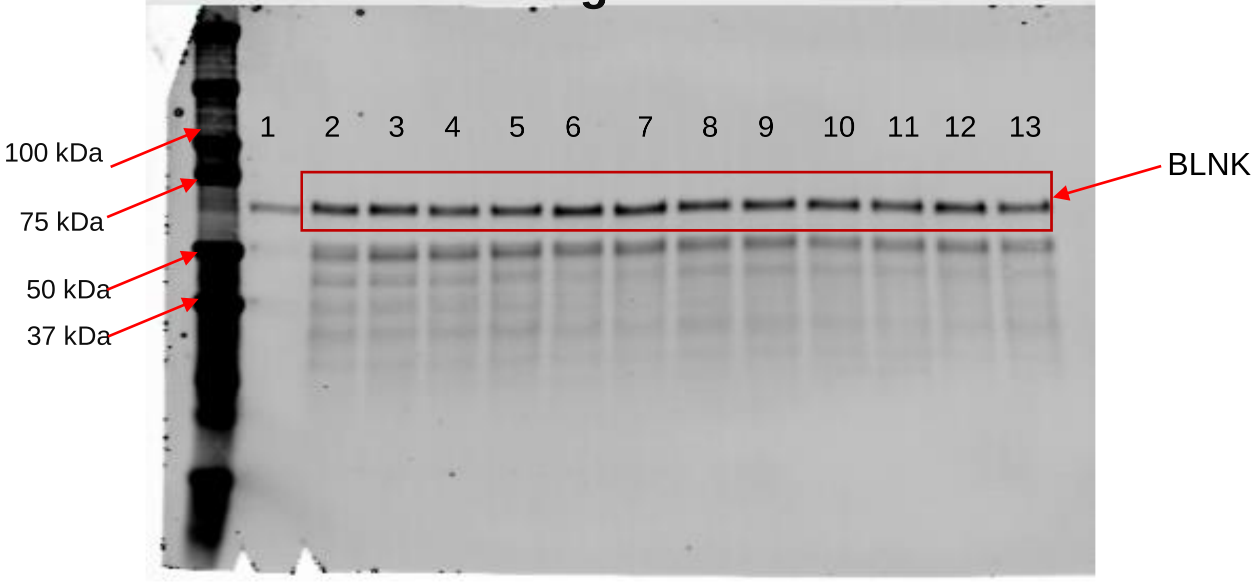

Figure 3A

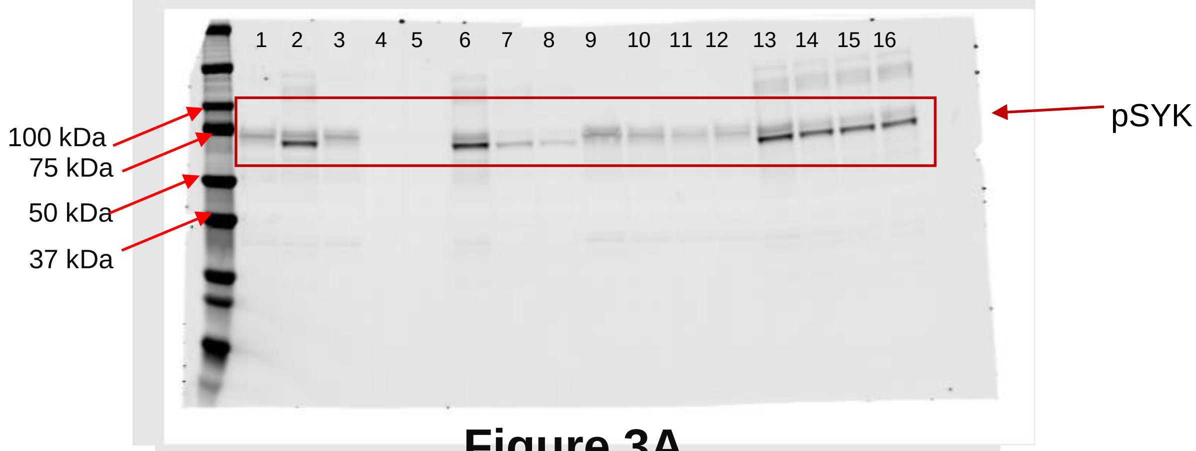

Figure 3A

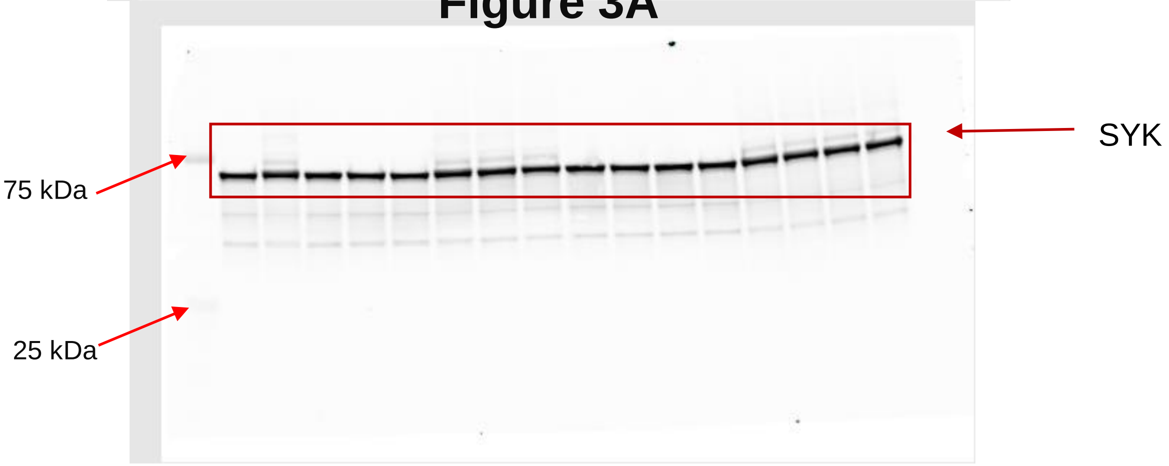

Figure 3A

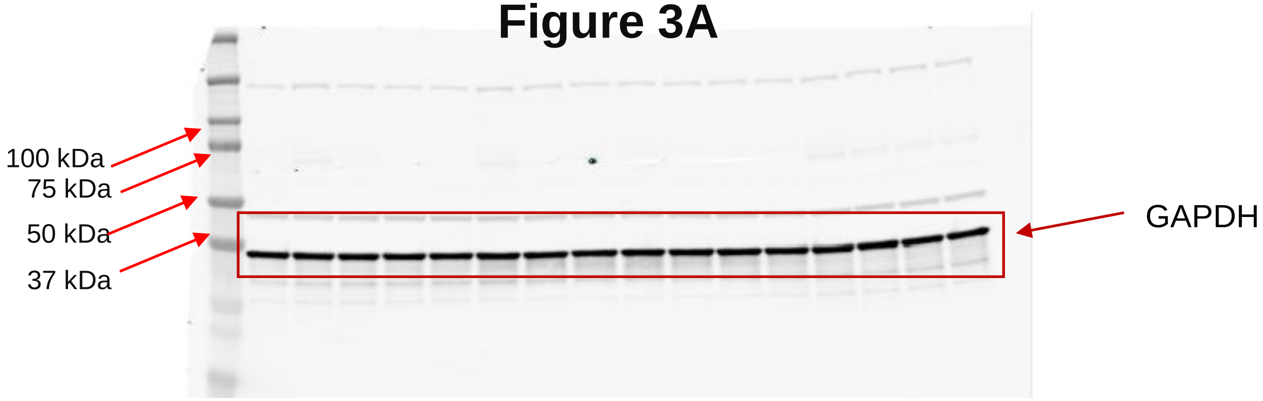

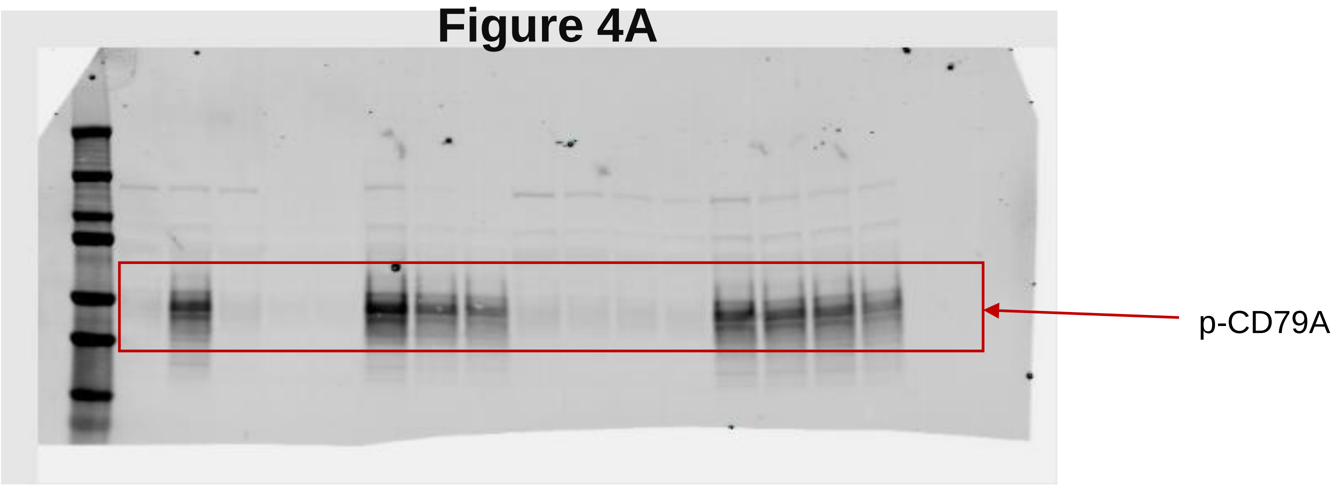
Figure 4A
p-CD79A

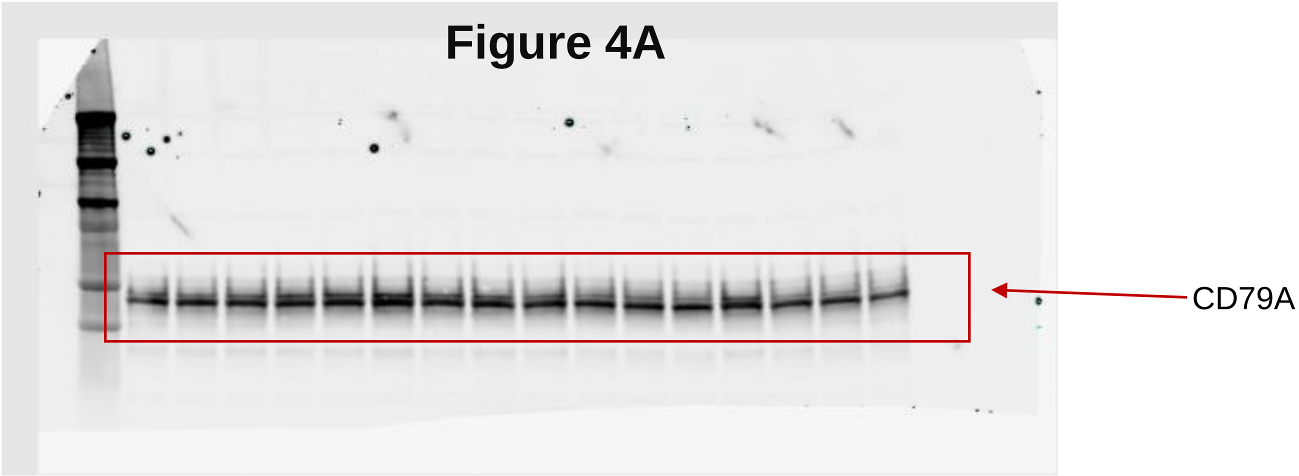
Figure 4A
CD79A

Figure 4A

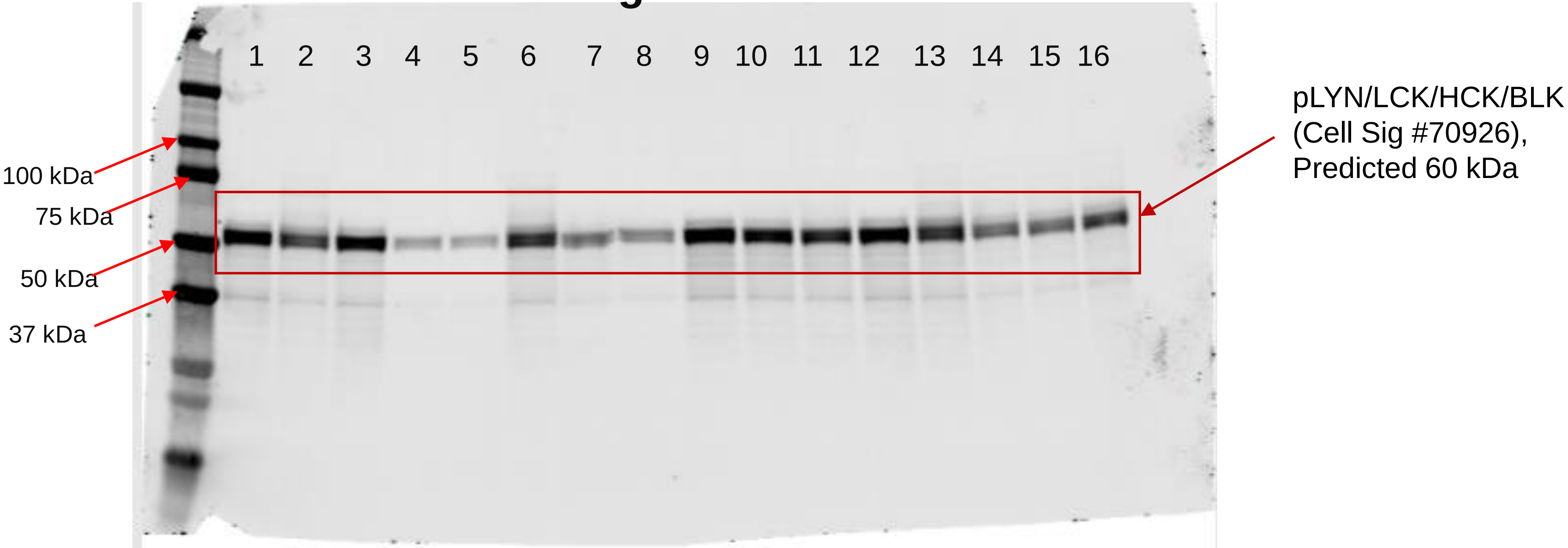

Figure 4A

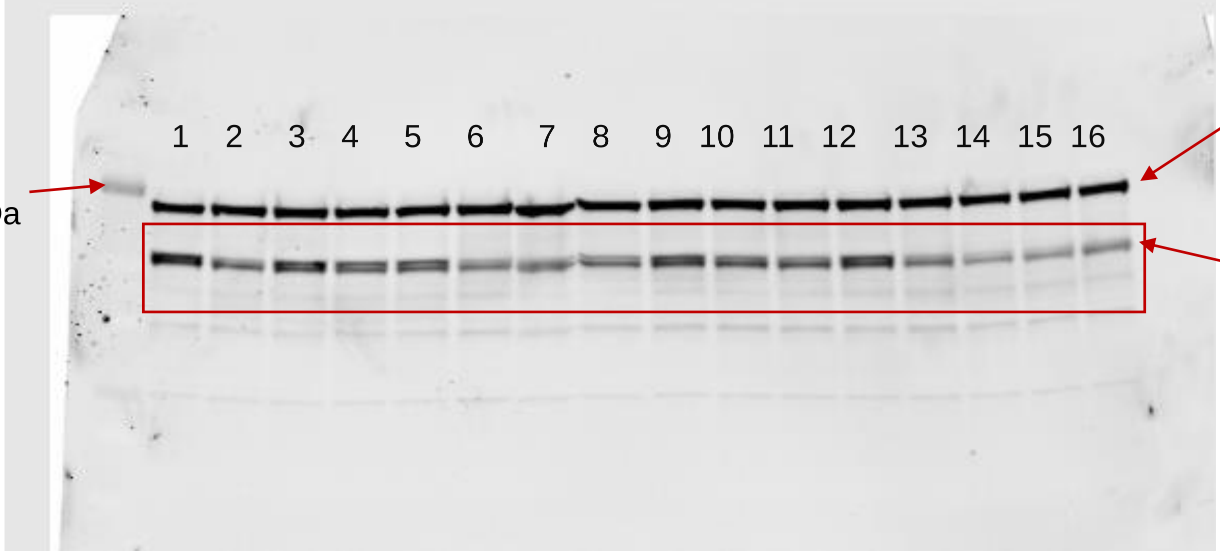

Figure 4A

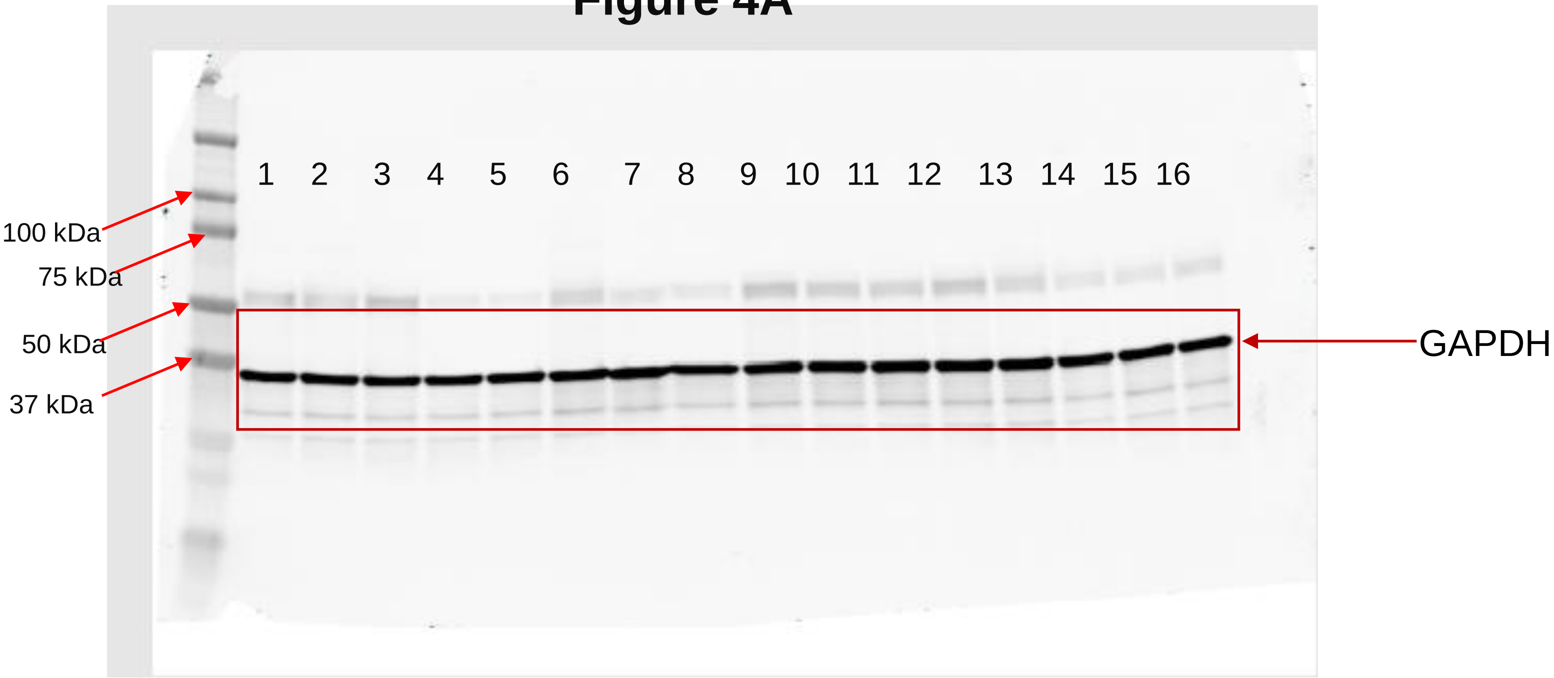

Figure 5

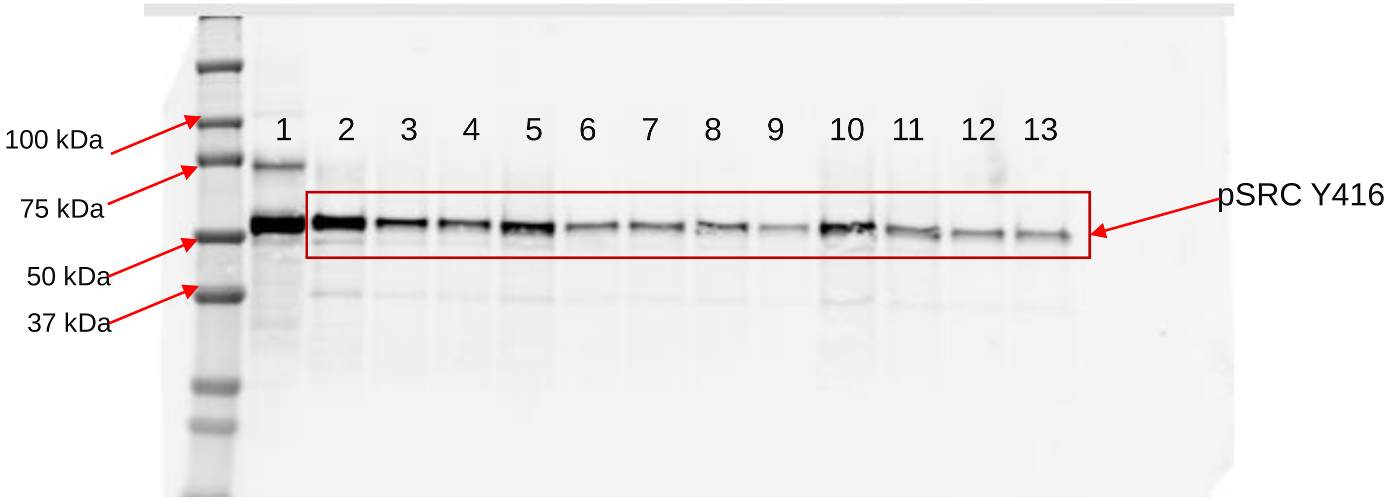

Figure 5

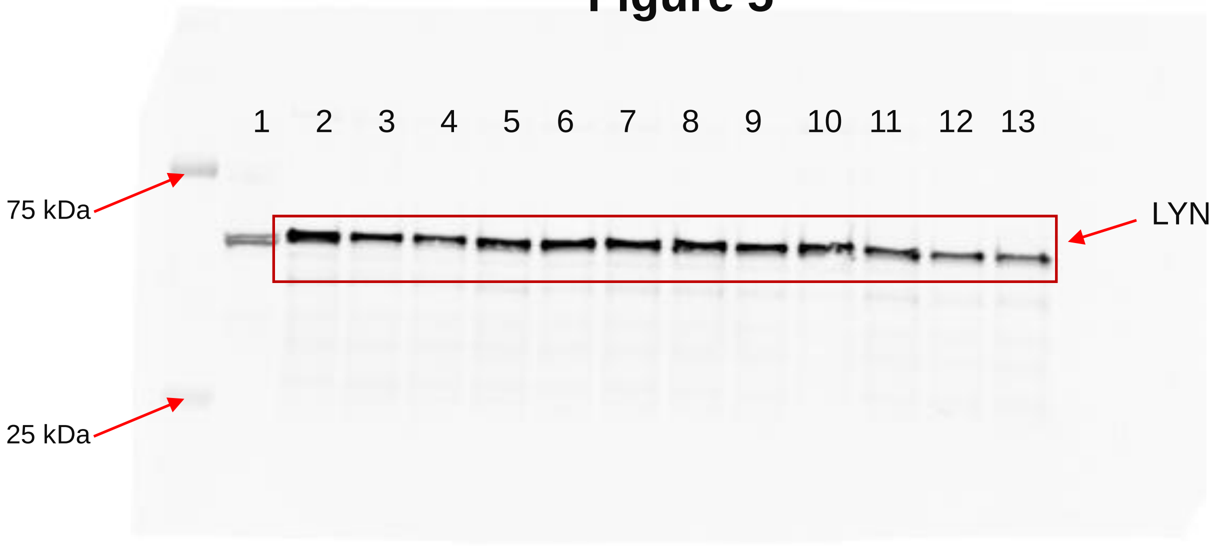

Figure 6A- SU-DHL-6

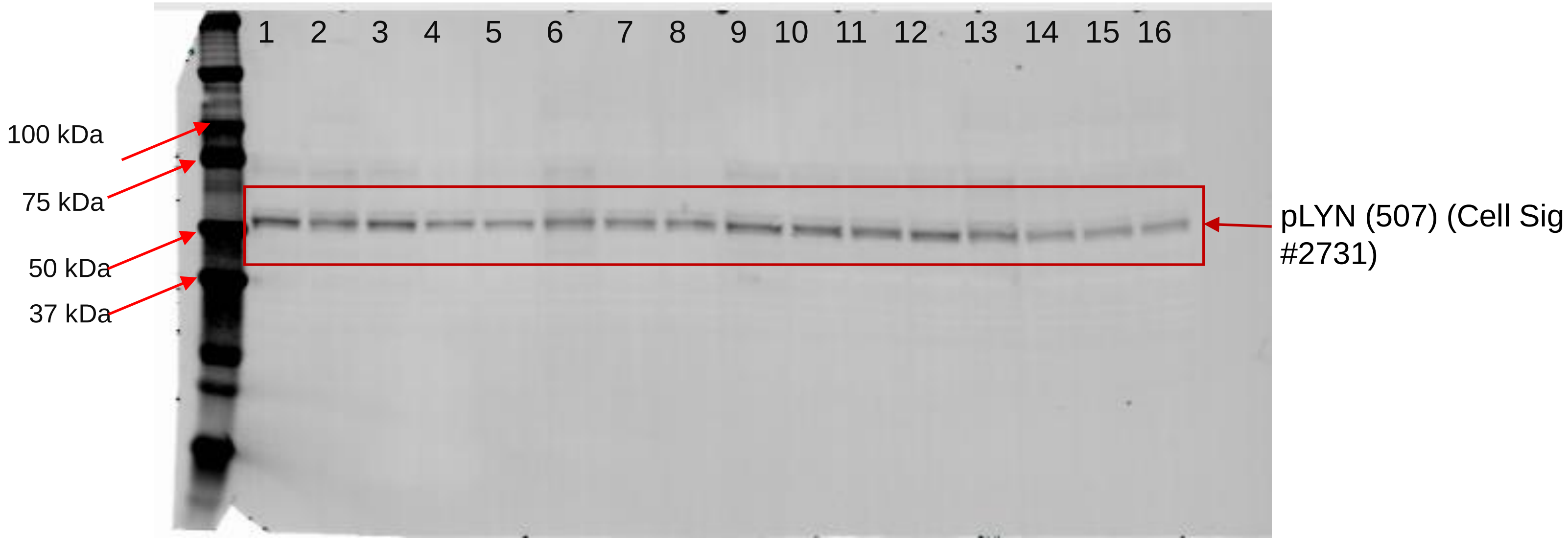

Figure 6A- SU-DHL-6

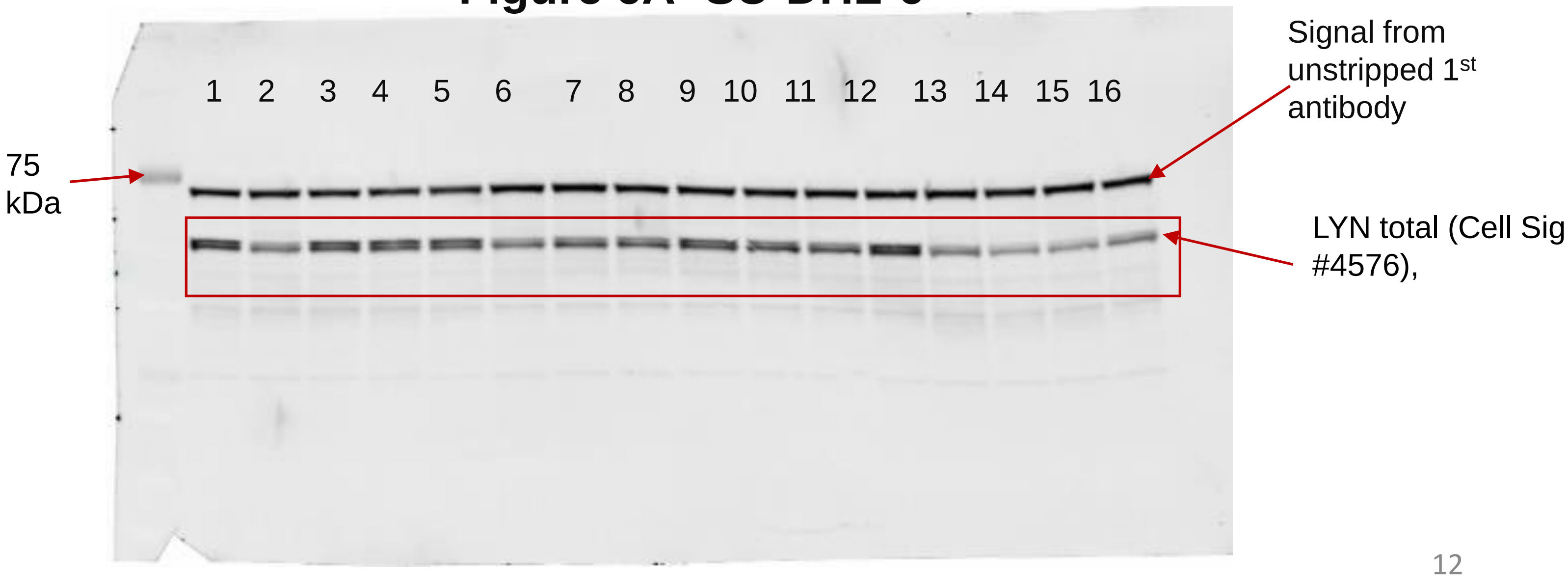

Figure 6A- JeKo-1

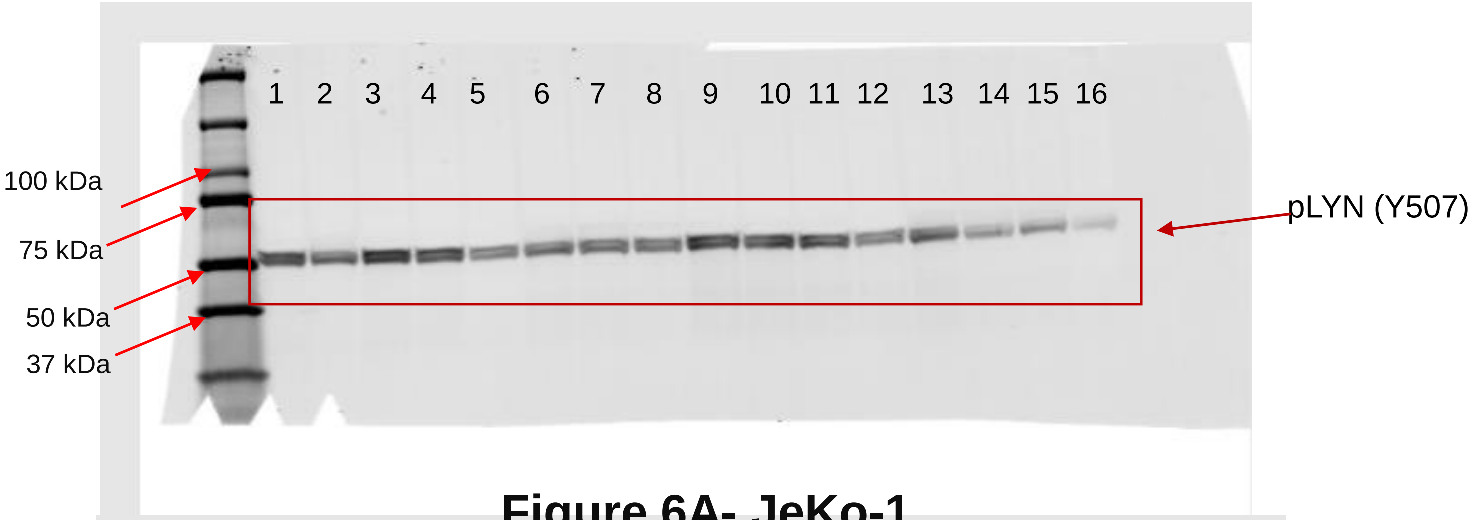

Figure 6A- JeKo-1

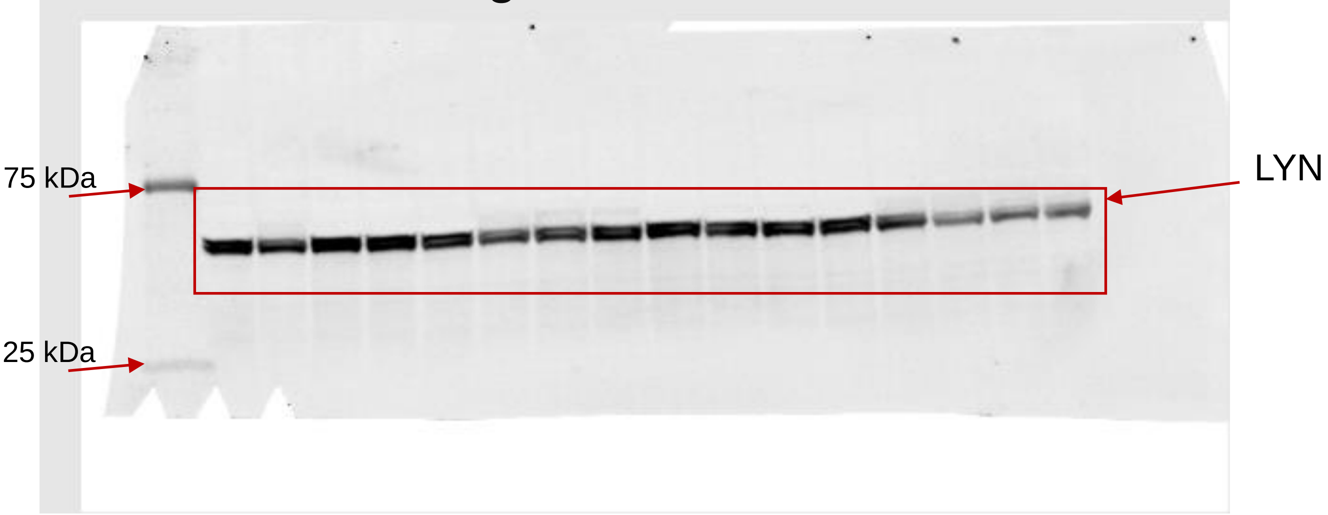

Figure 6A- RL

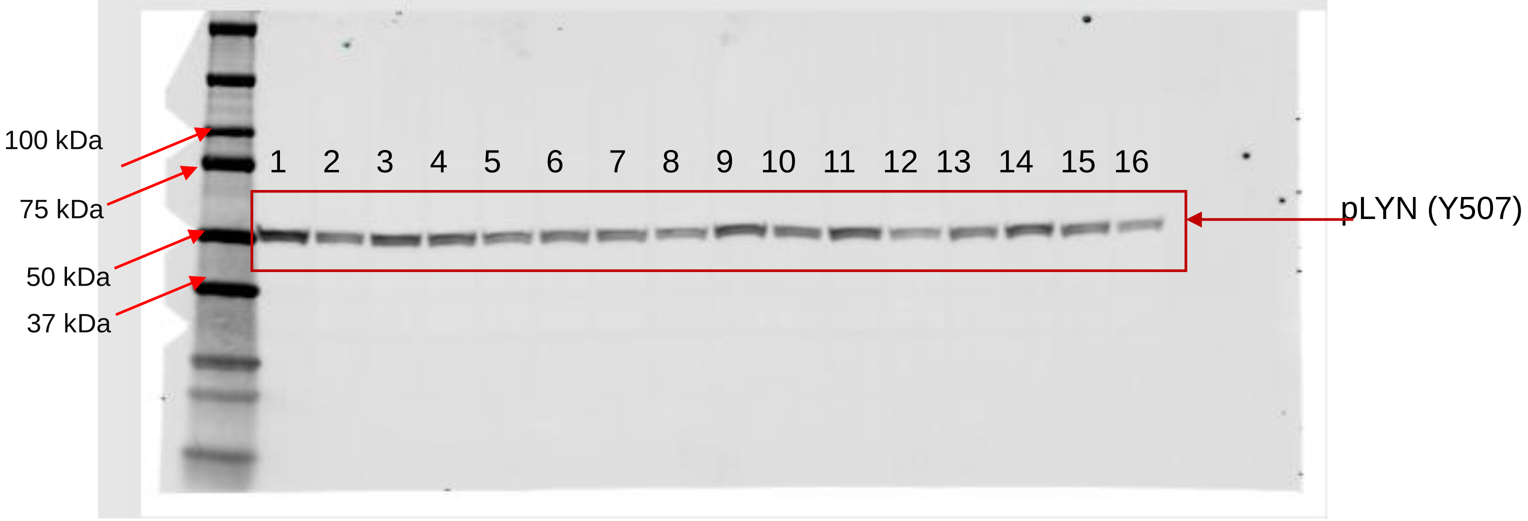

Figure 6A- RL

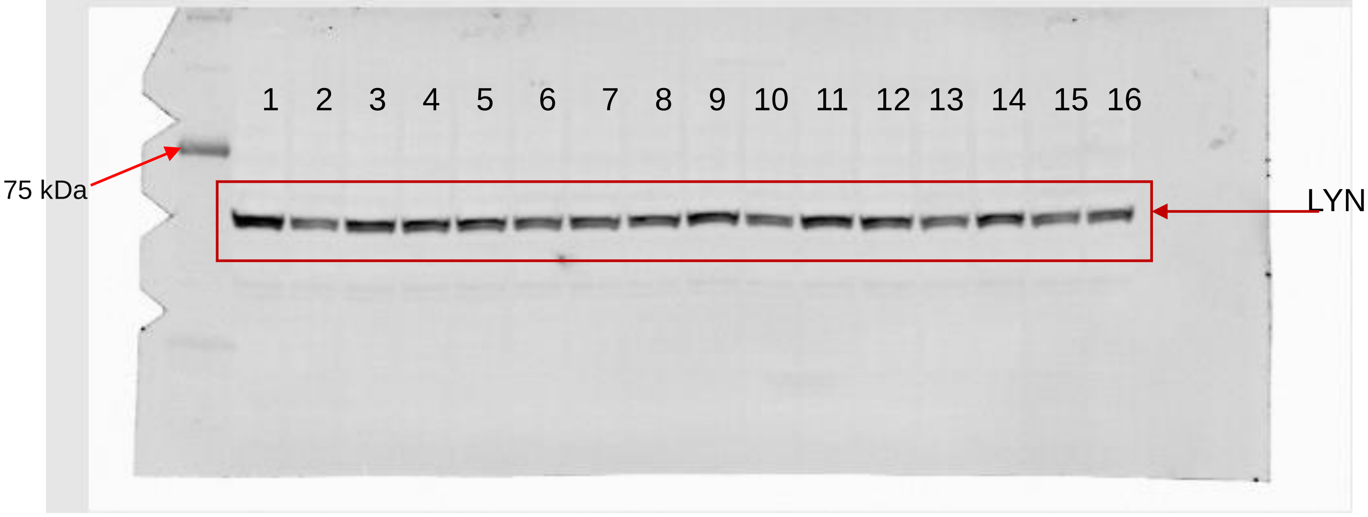

Supplementary Figure 1A

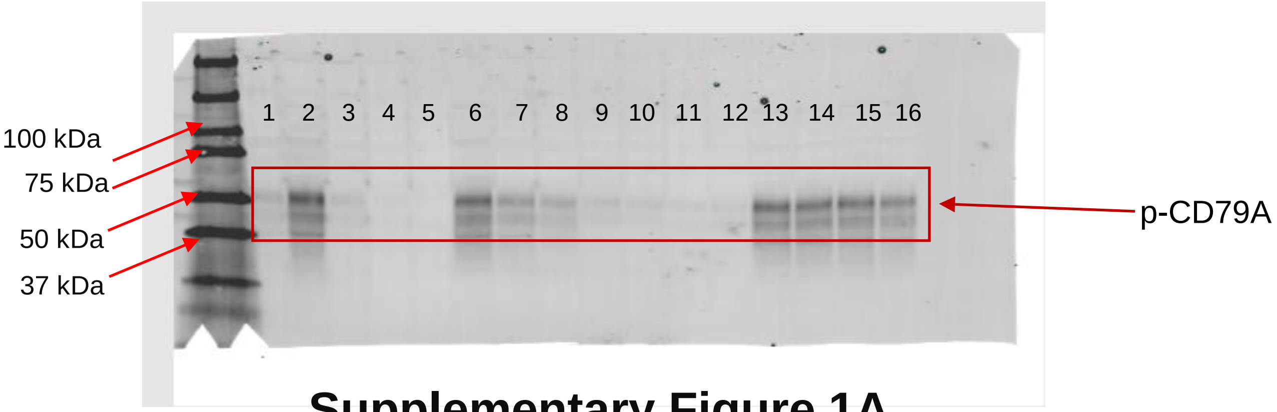

Supplementary Figure 1A

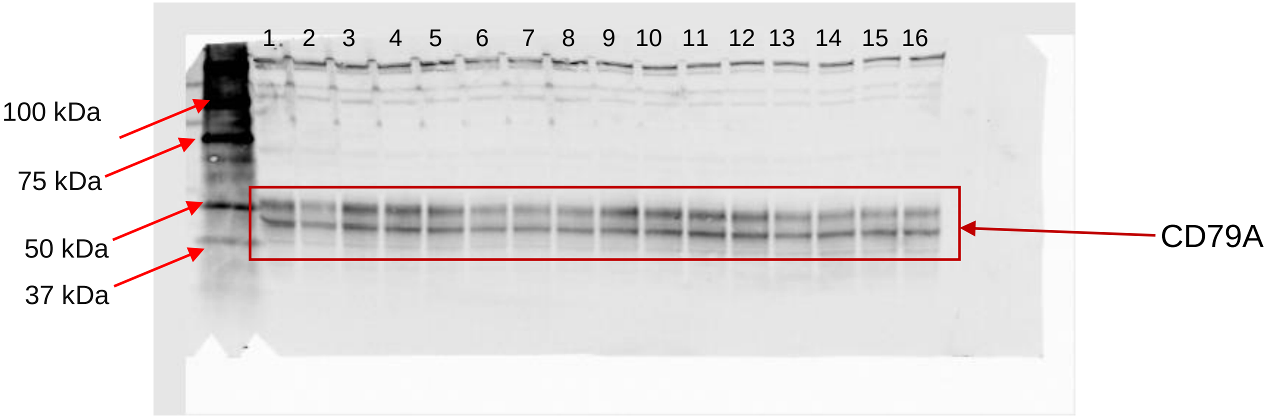

Supplementary Figure 1A

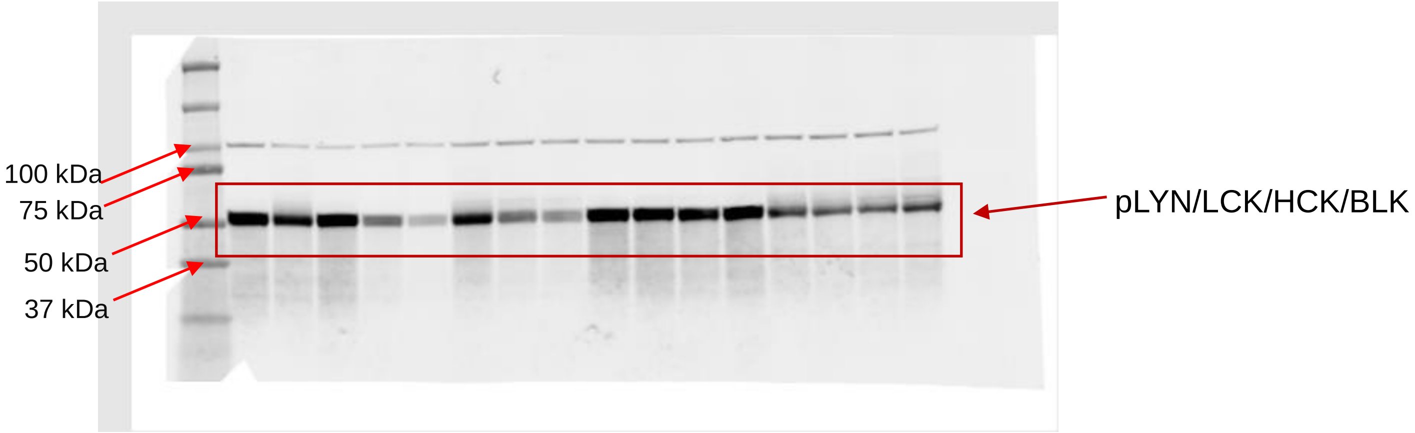

Supplementary Figure 1A

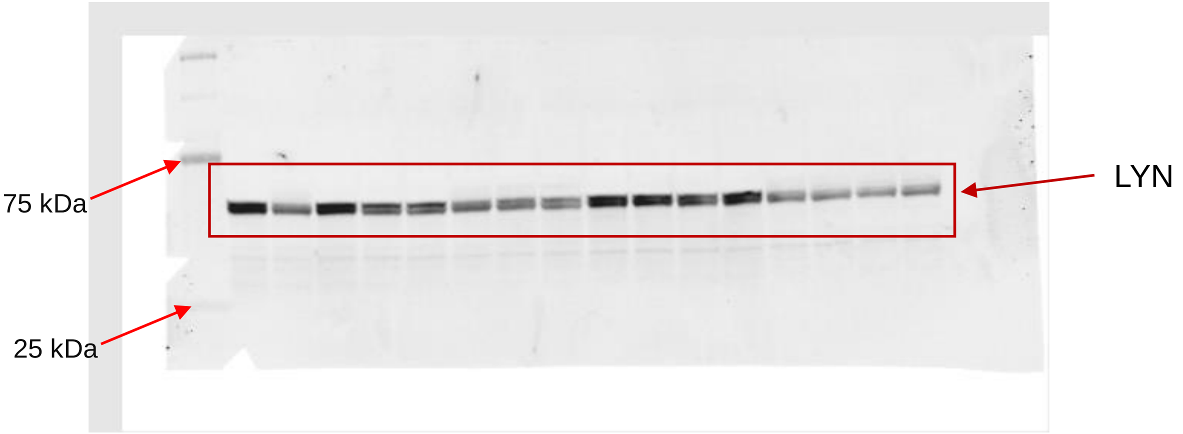

Supplementary Figure 1A

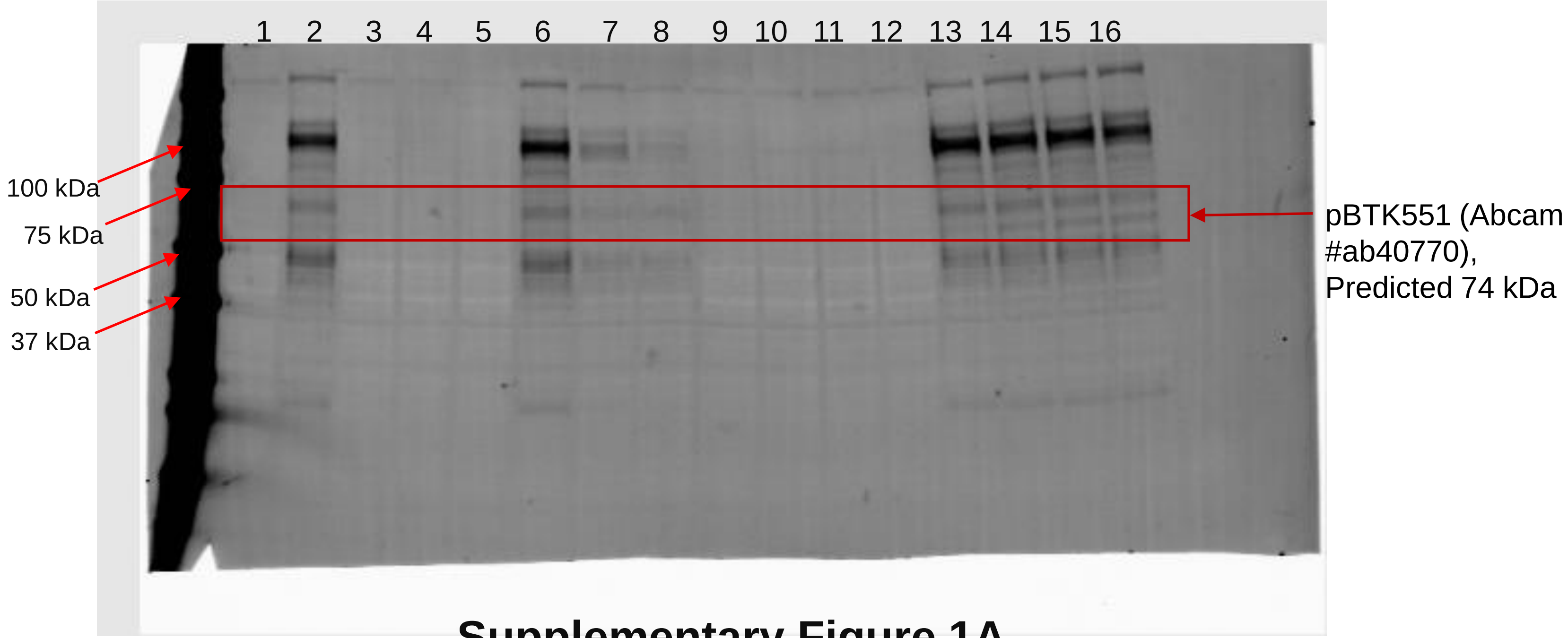

Supplementary Figure 1A

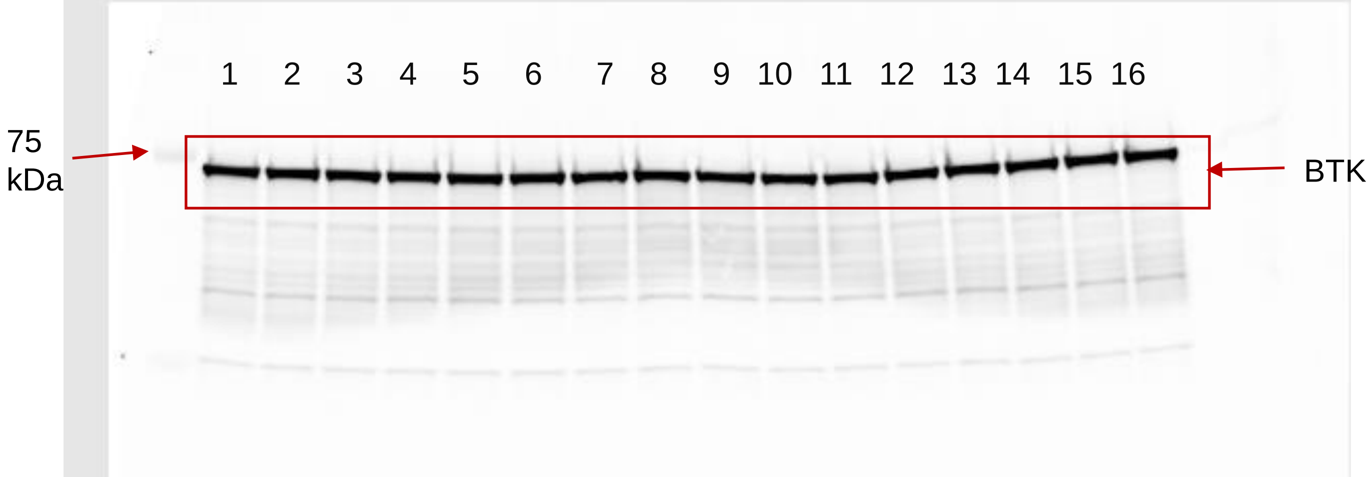

Supplementary Figure 1A

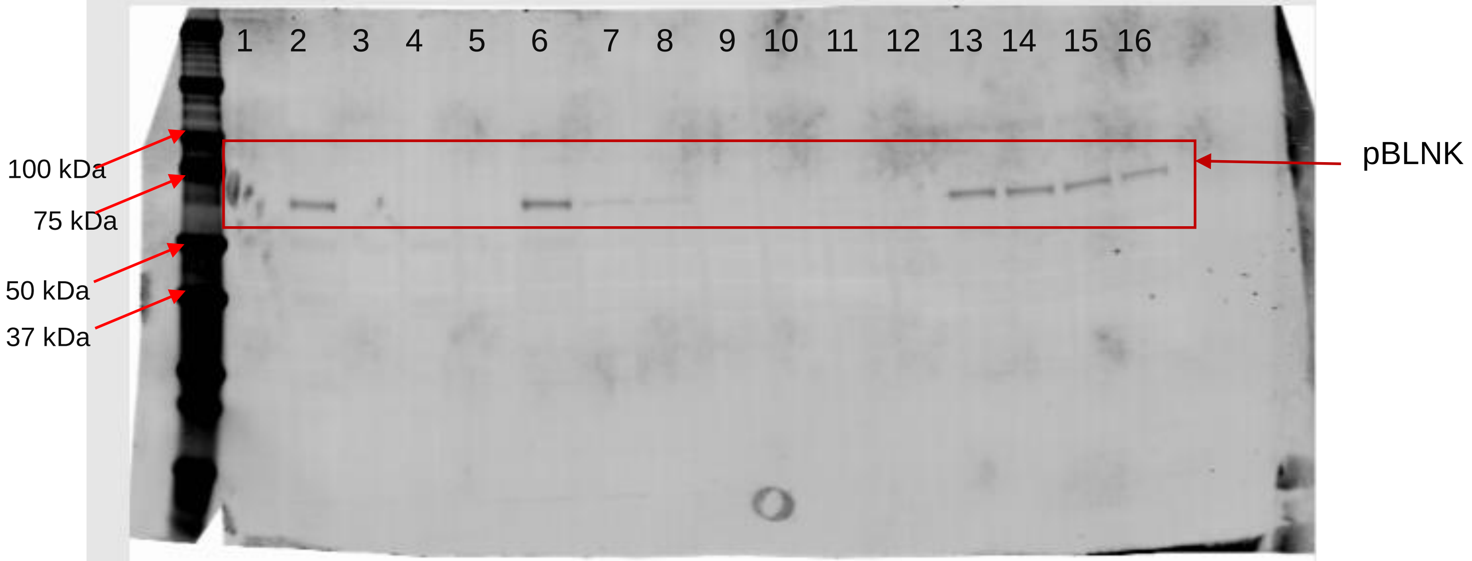

Supplementary Figure 1A

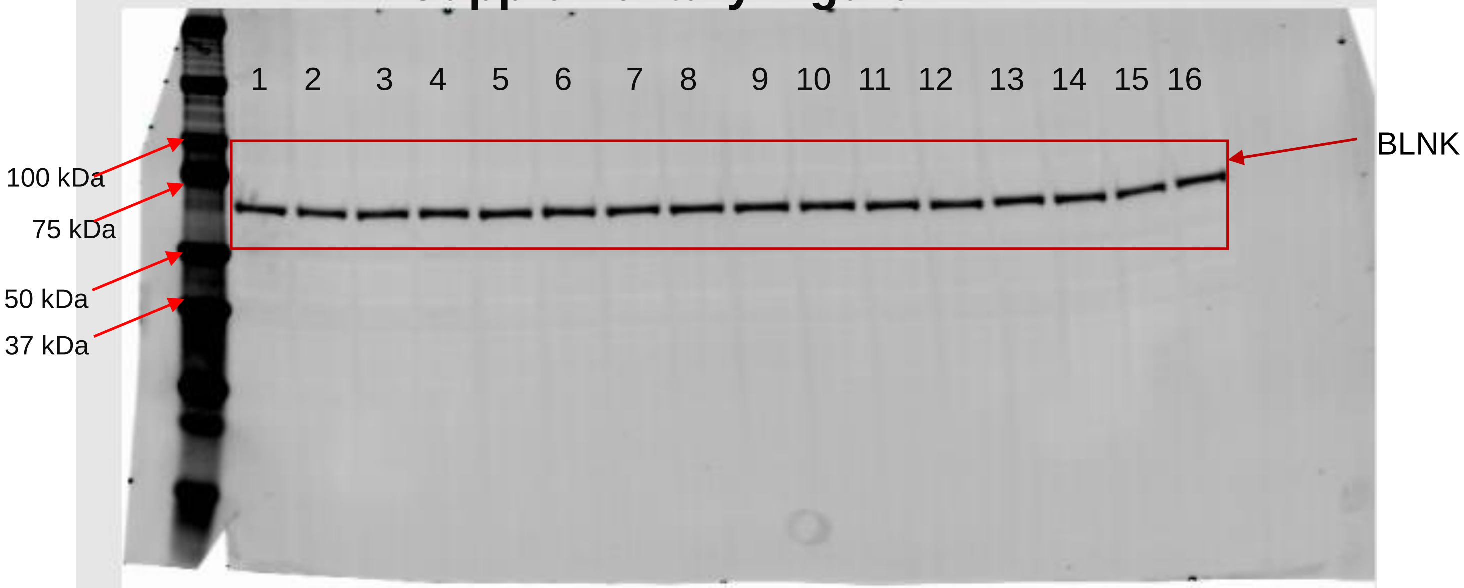

Supplementary Figure 1A

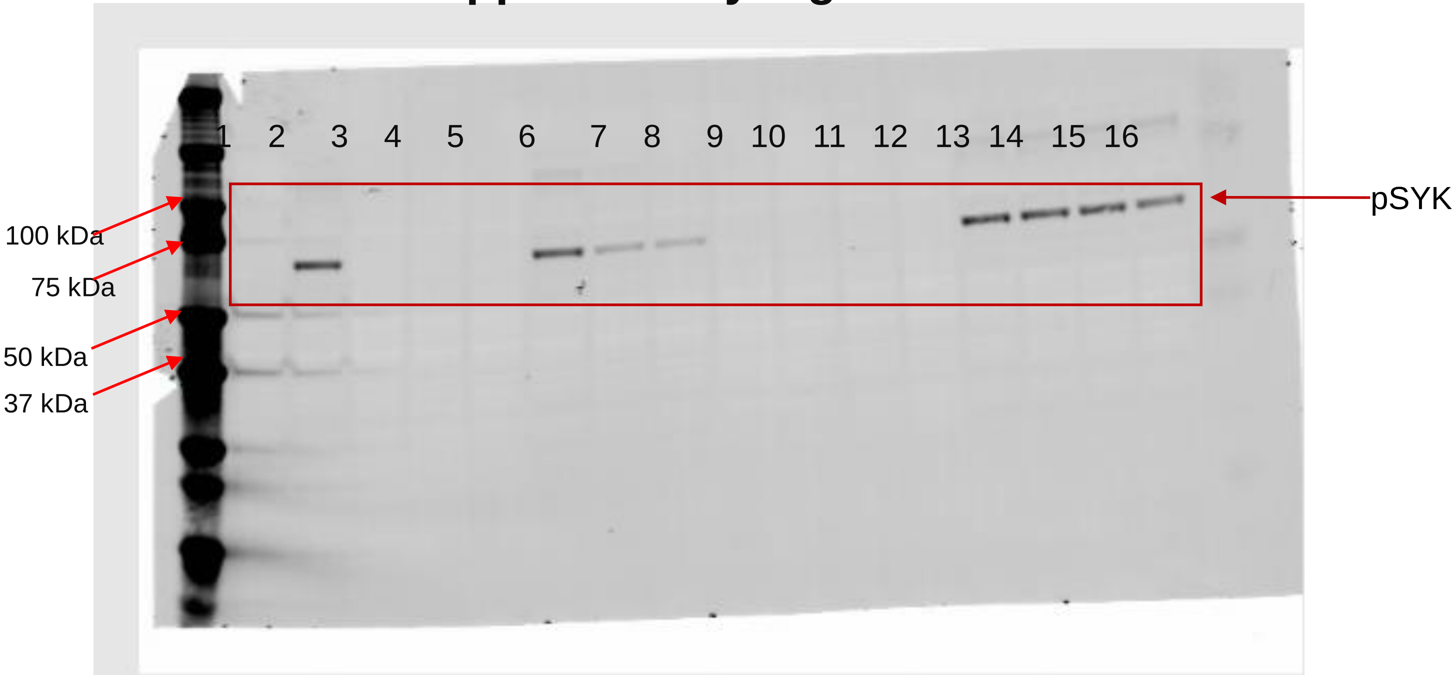

Supplementary Figure 1A

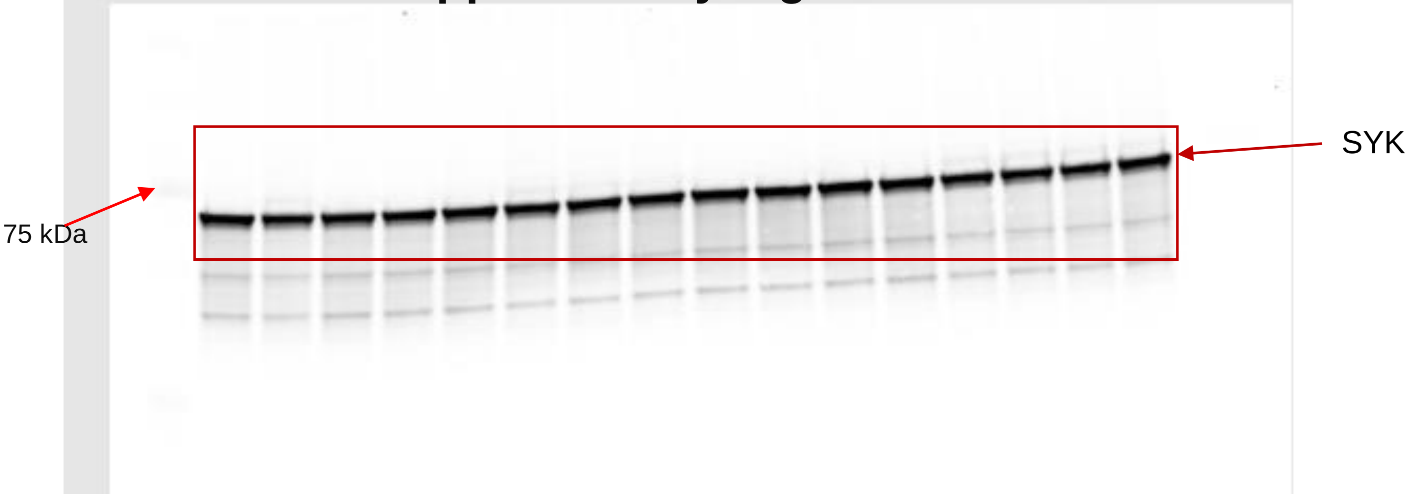

Supplementary Figure 1A

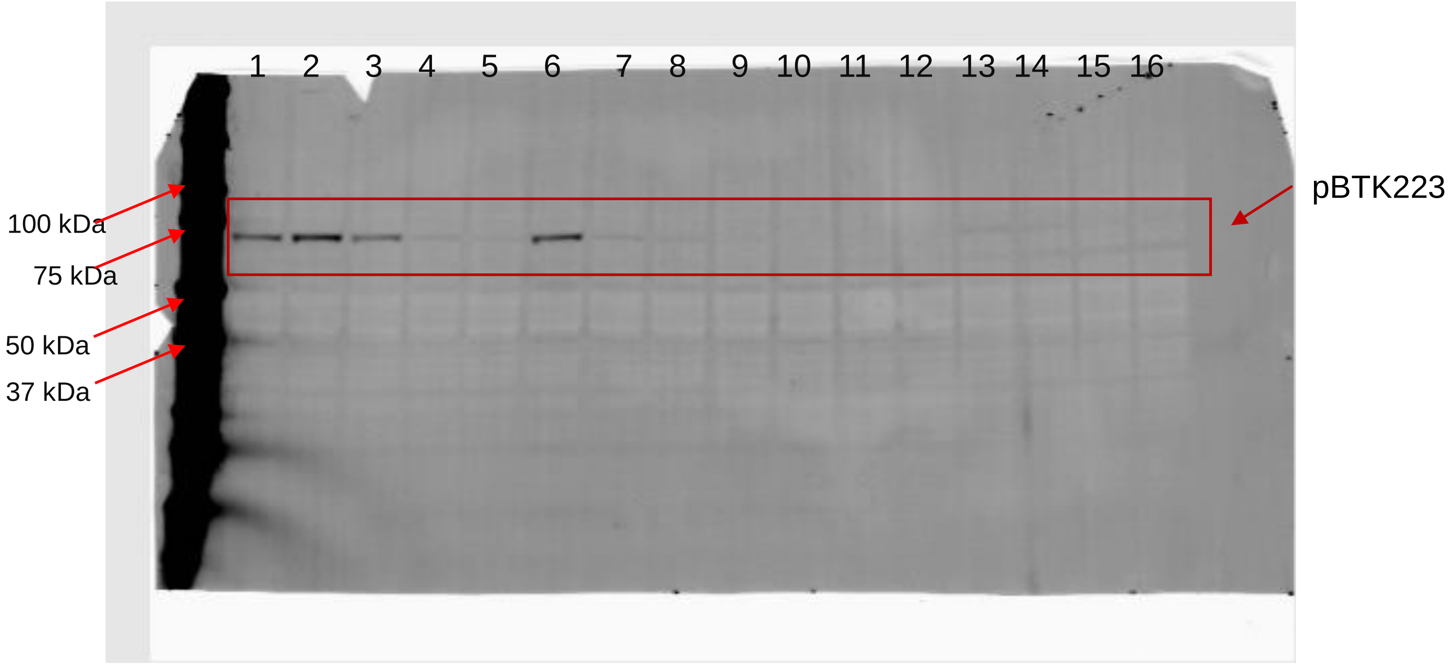

Supplementary Figure 1A

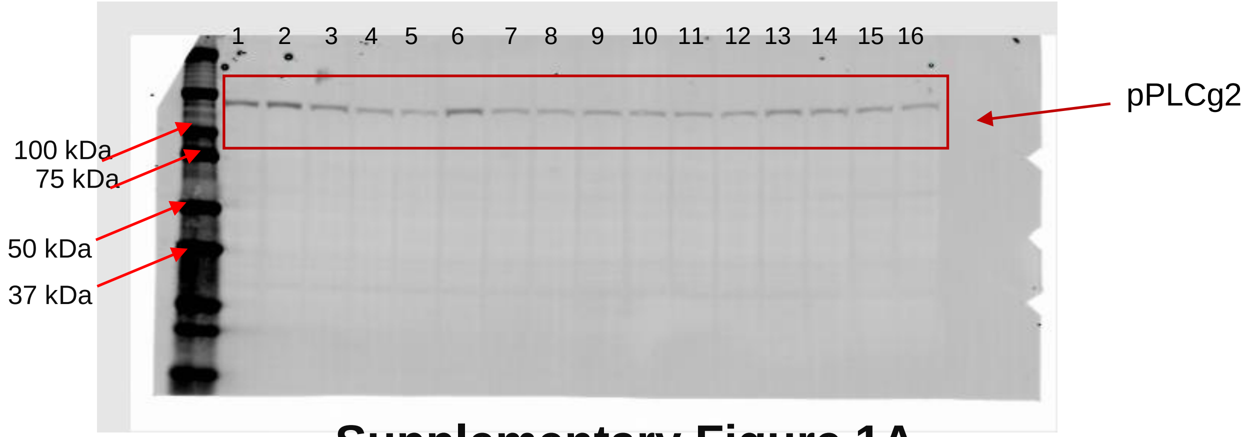

Supplementary Figure 1A

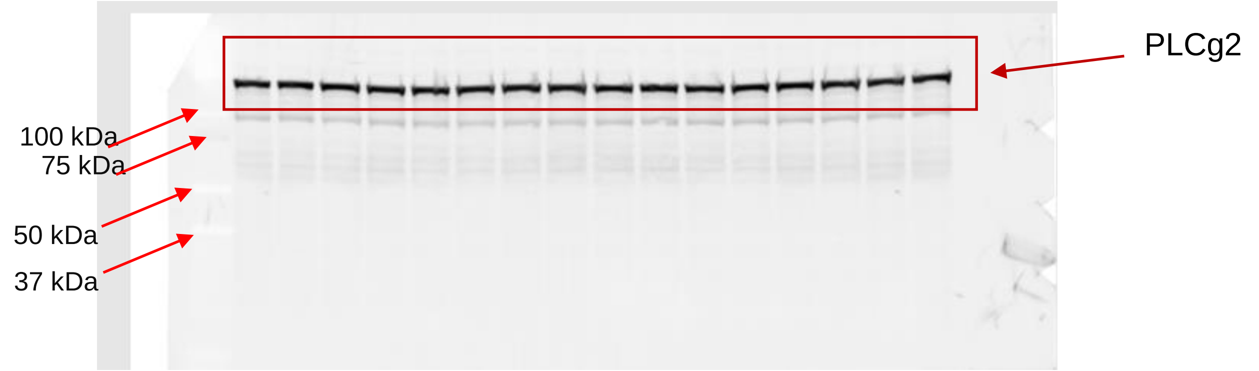

Supplementary Figure 1A

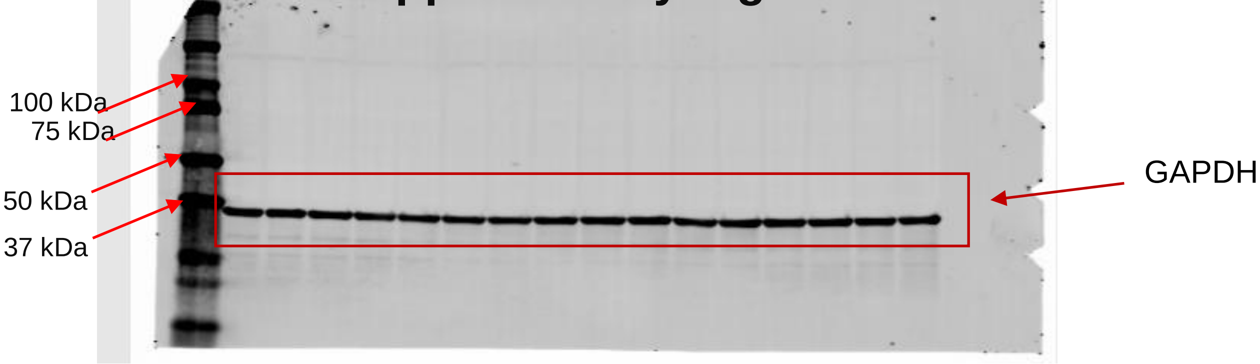

Supplementary figure 1B

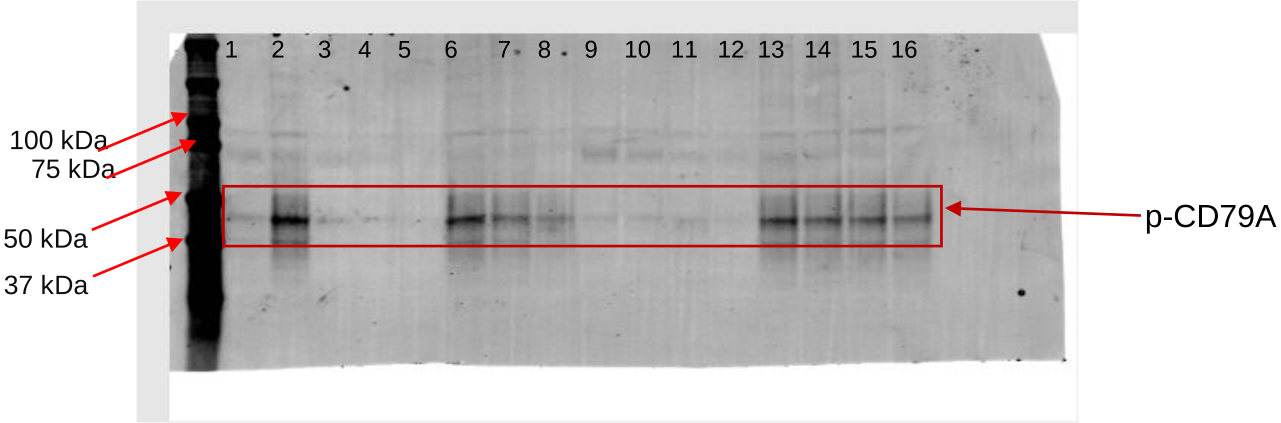

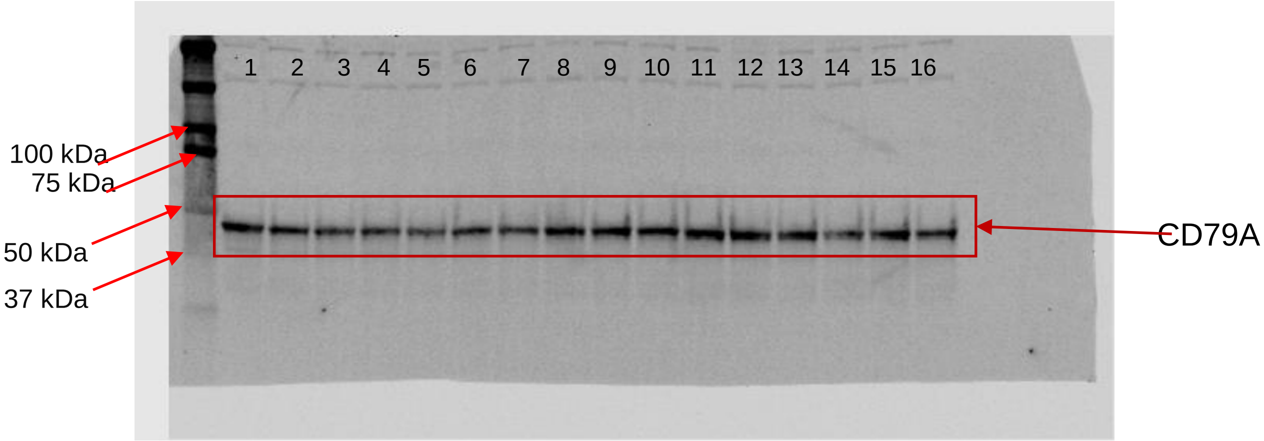

Supplementary figure 1B

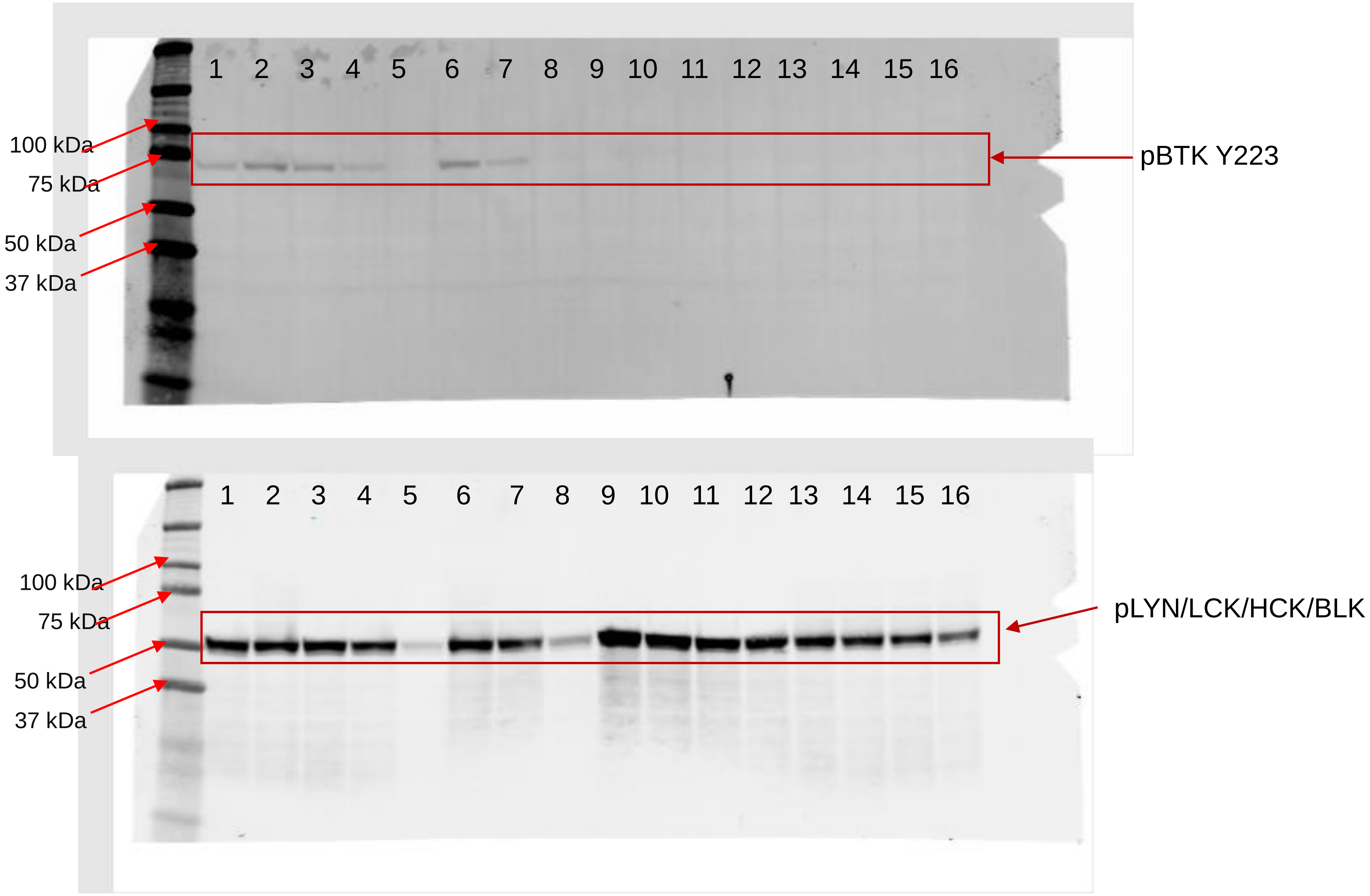

Supplementary figure 1B

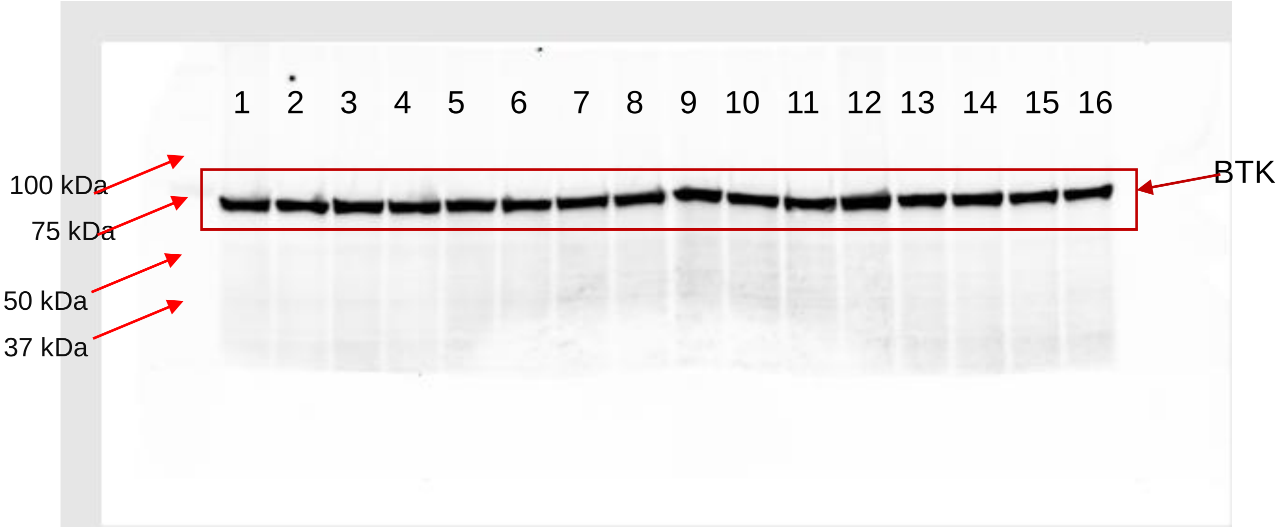

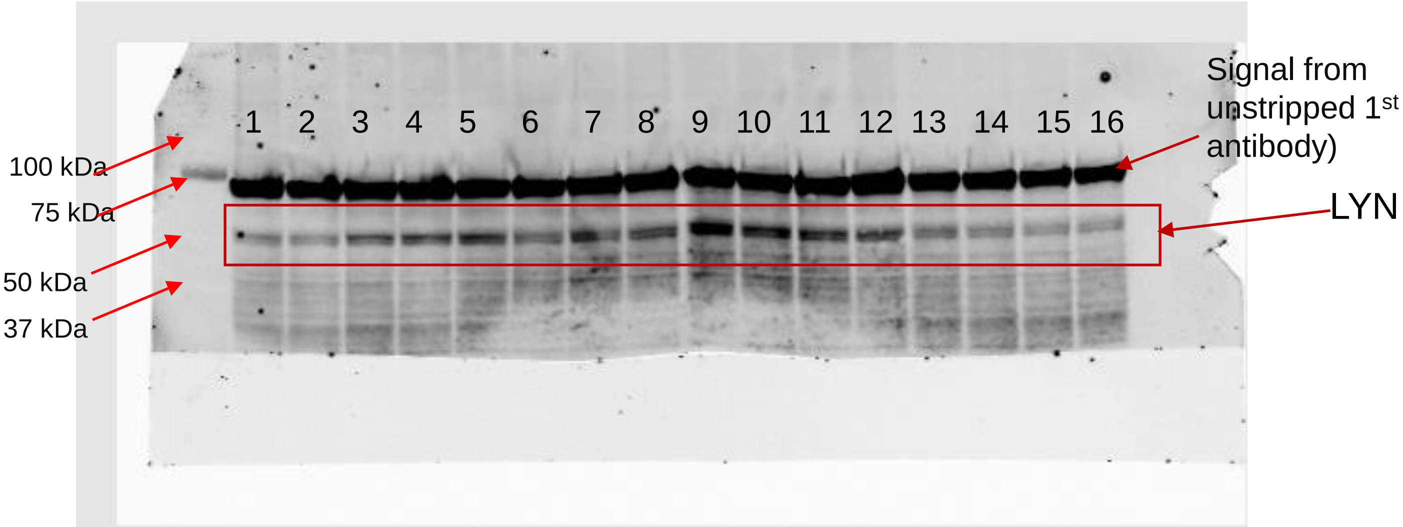

Supplementary figure 1B

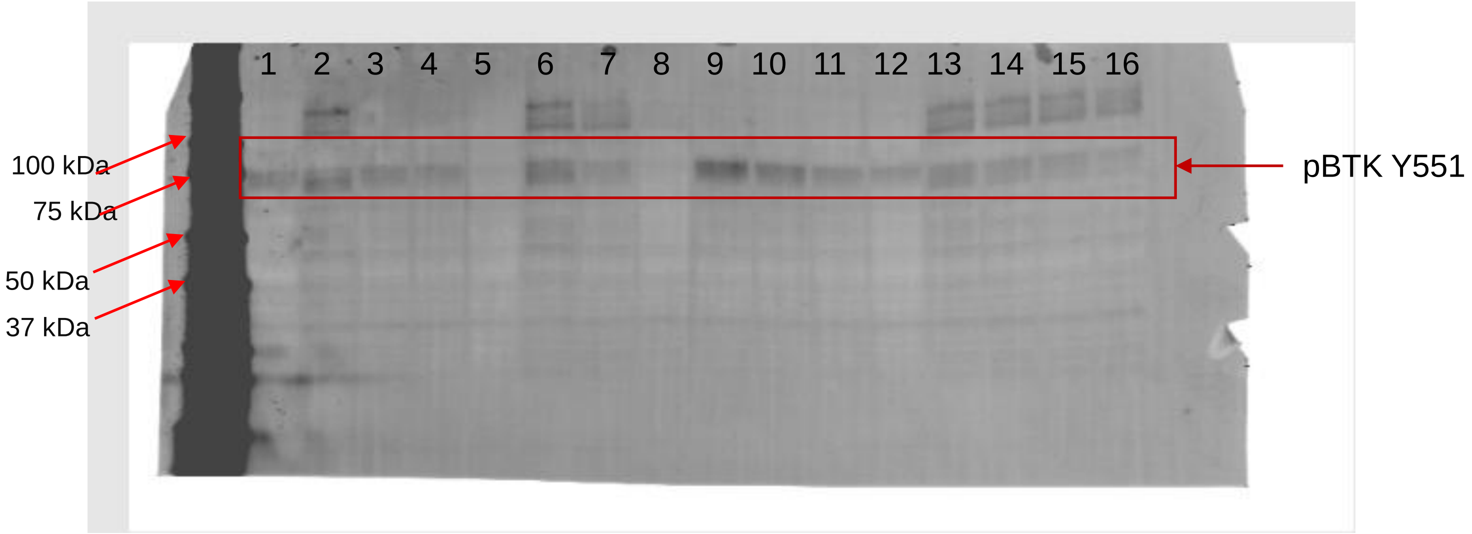

Supplementary figure 1B

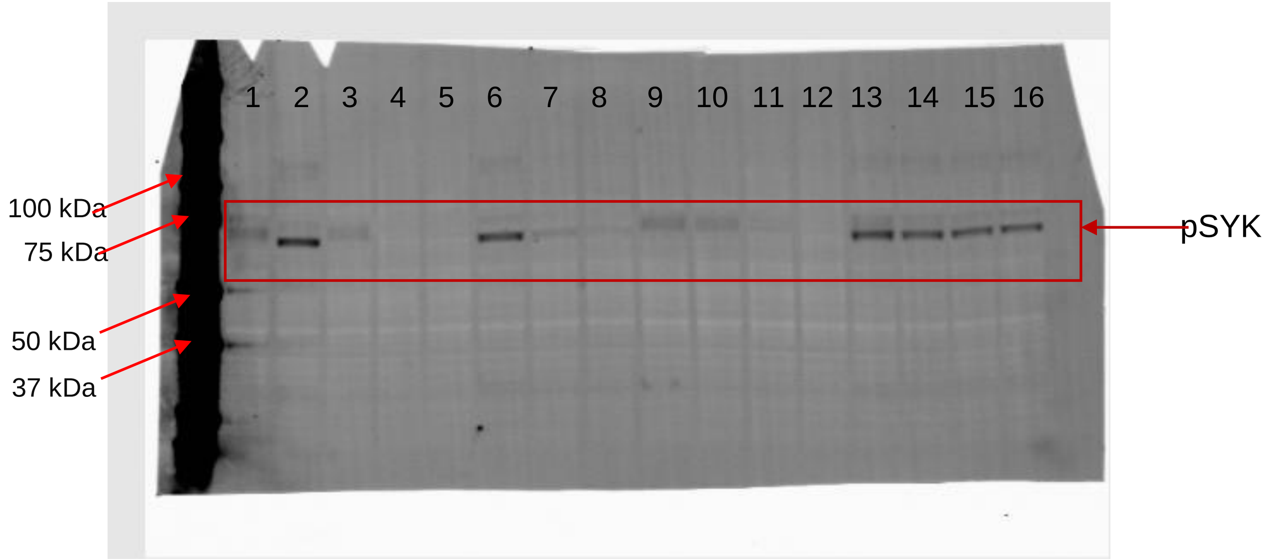

Supplementary figure 1B

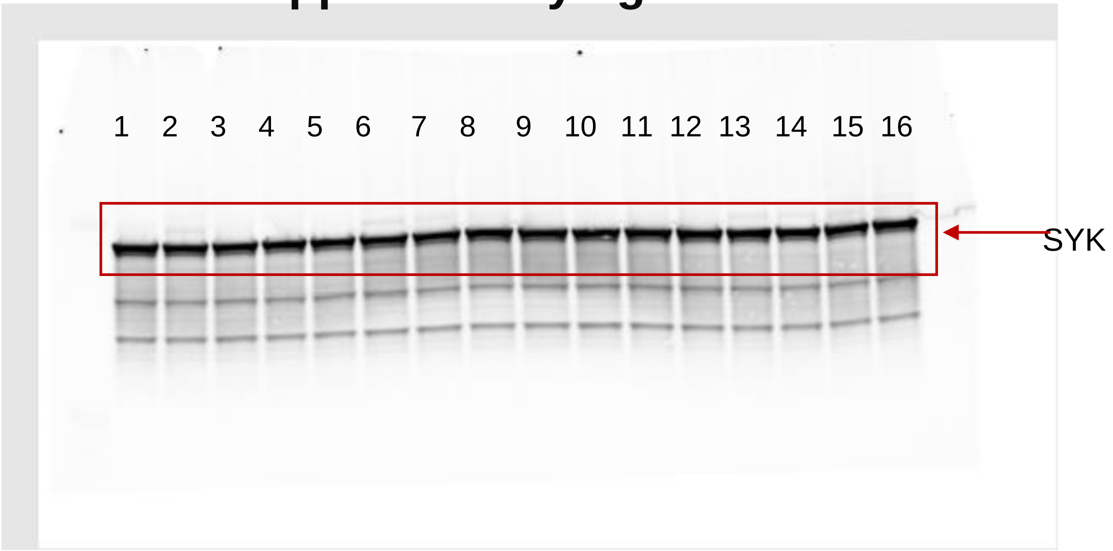

Supplementary figure 1B

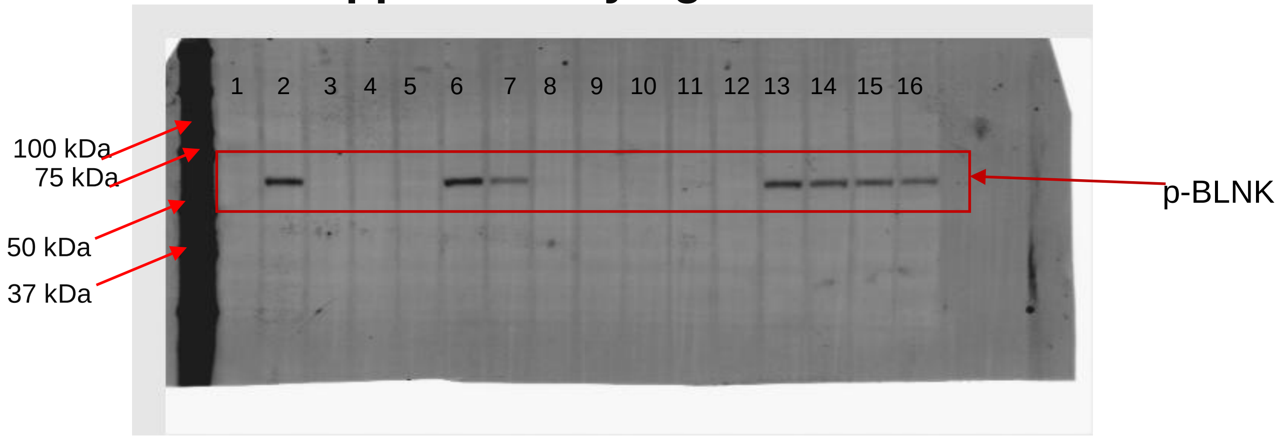

Supplementary figure 1B

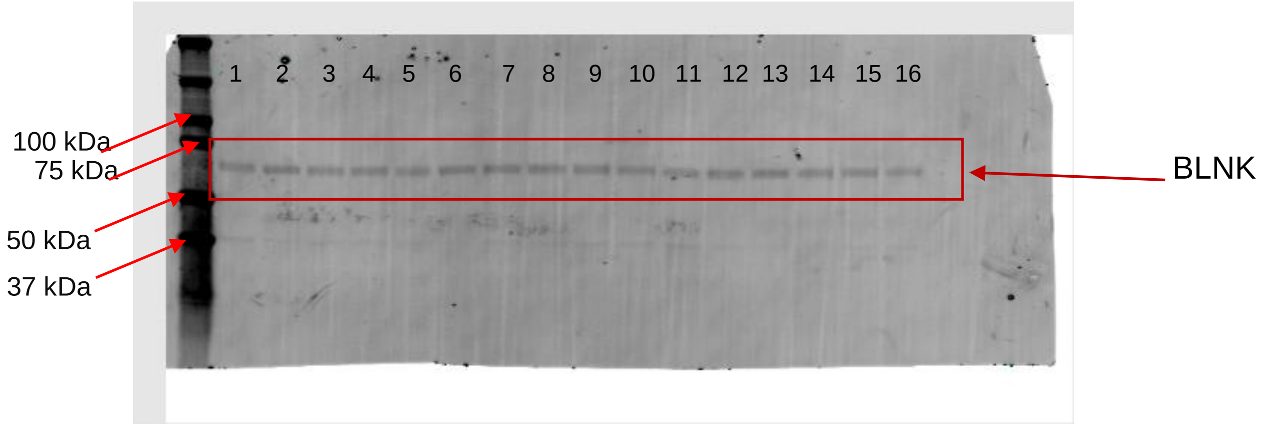

Supplementary figure 1B

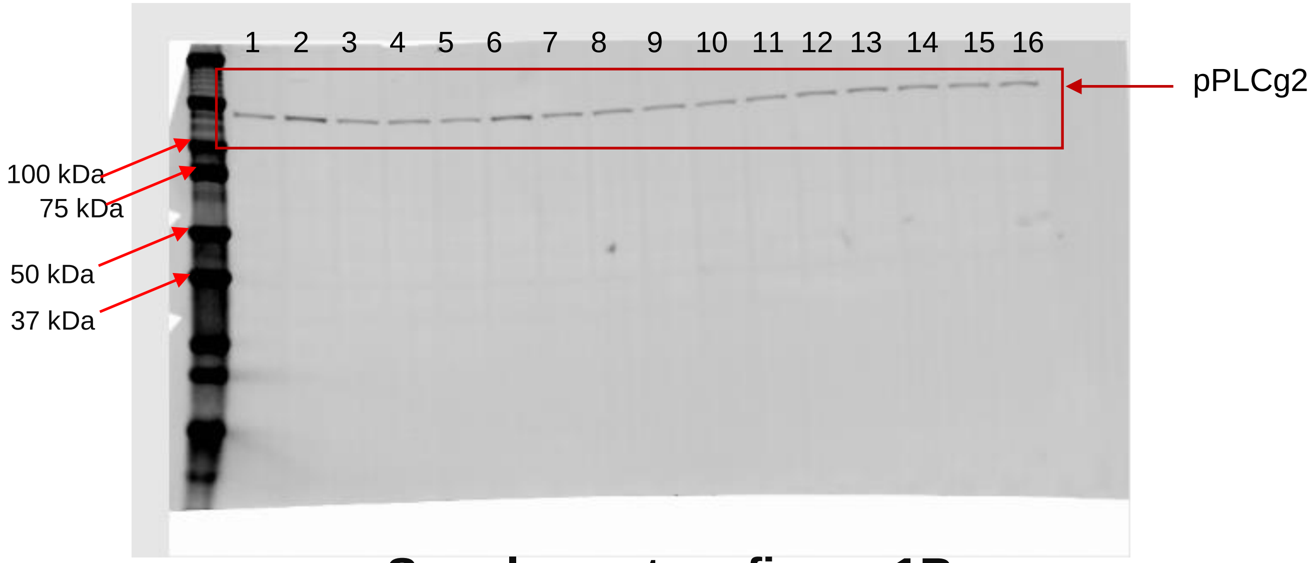

Supplementary figure 1B

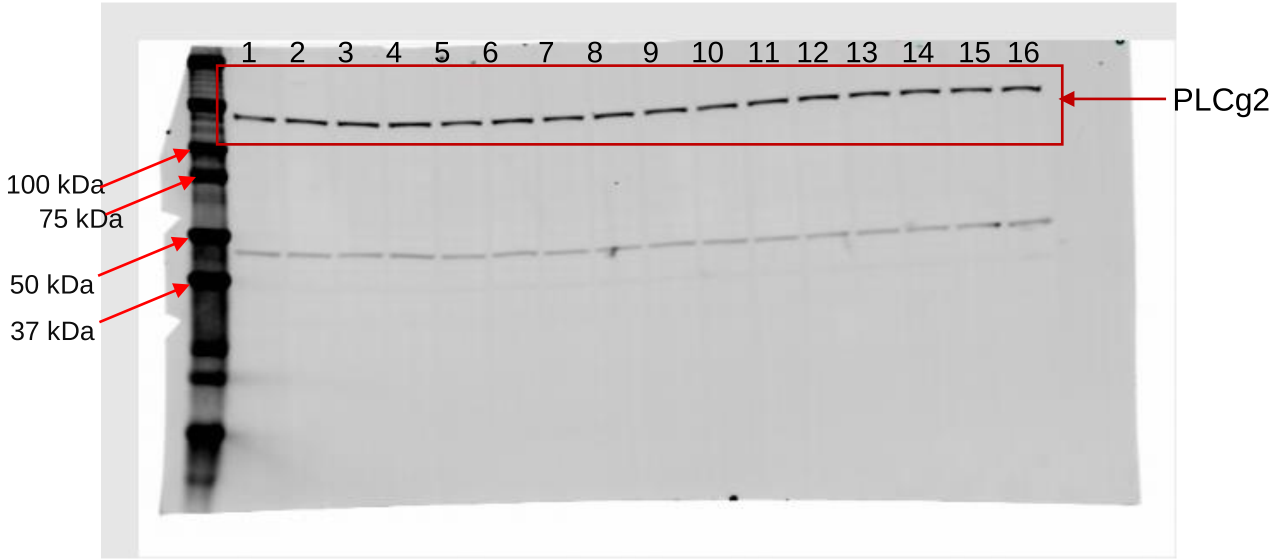

Supplementary figure 1B

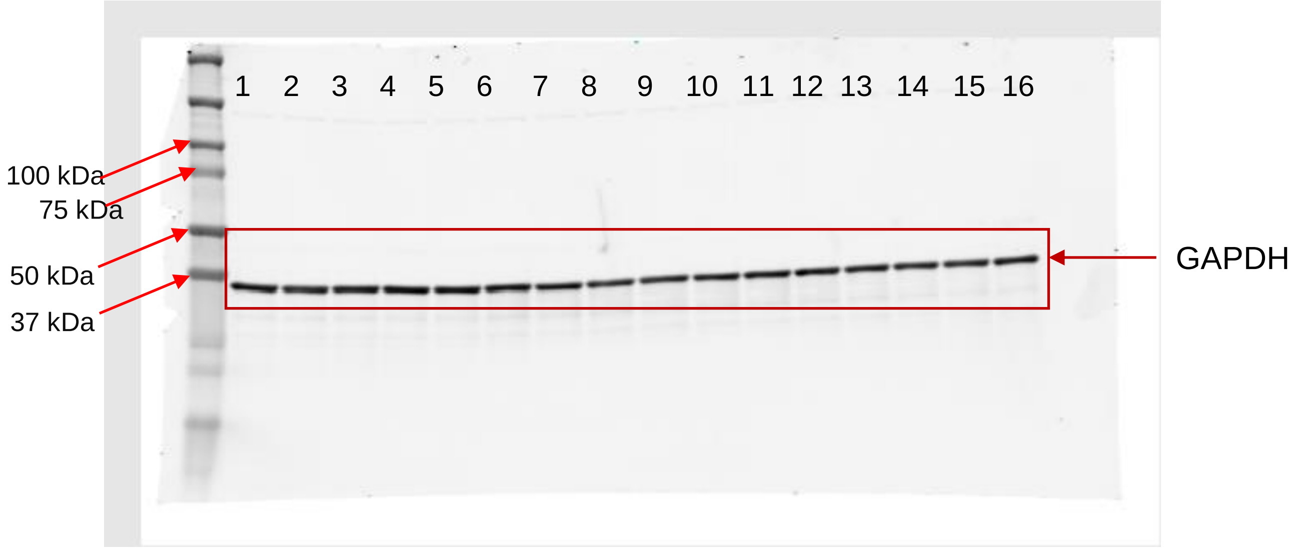

Supplementary figure 2A

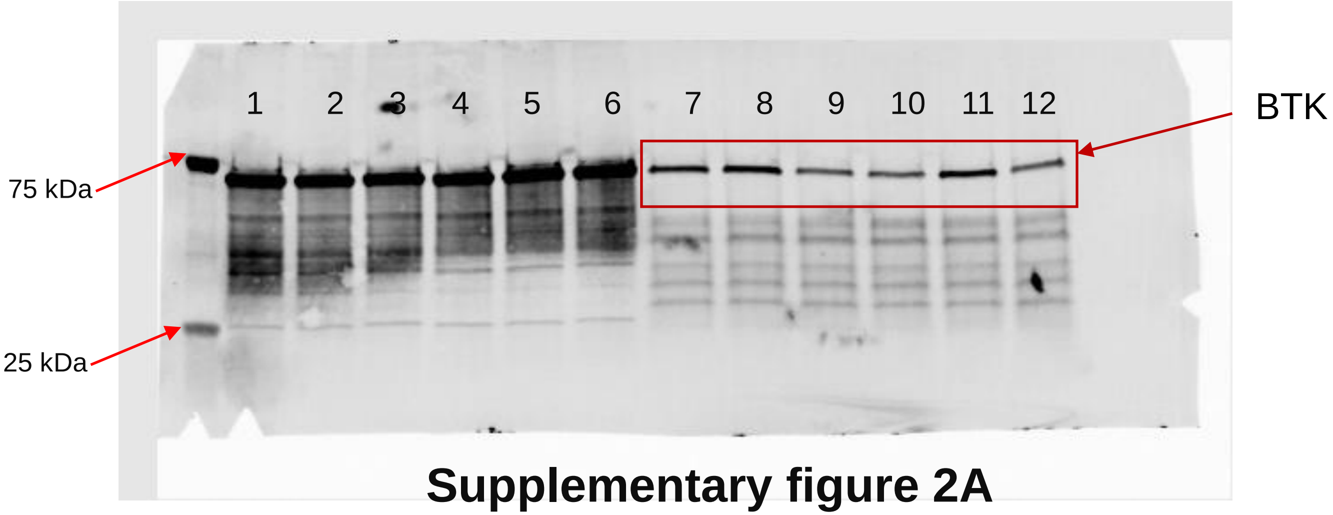

Supplementary figure 2A

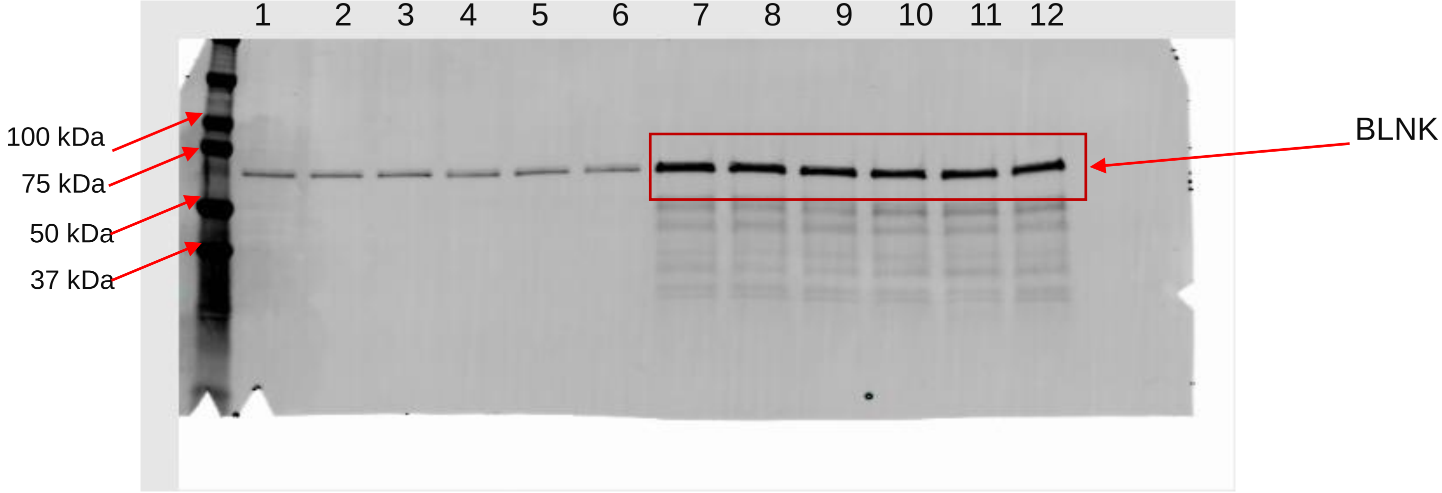

Supplementary figure 2B

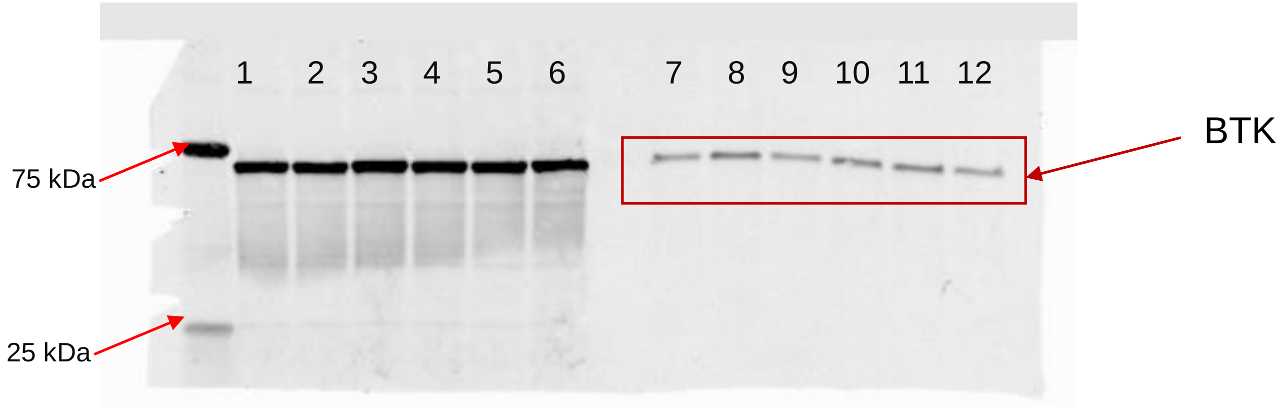

Supplementary figure 2B

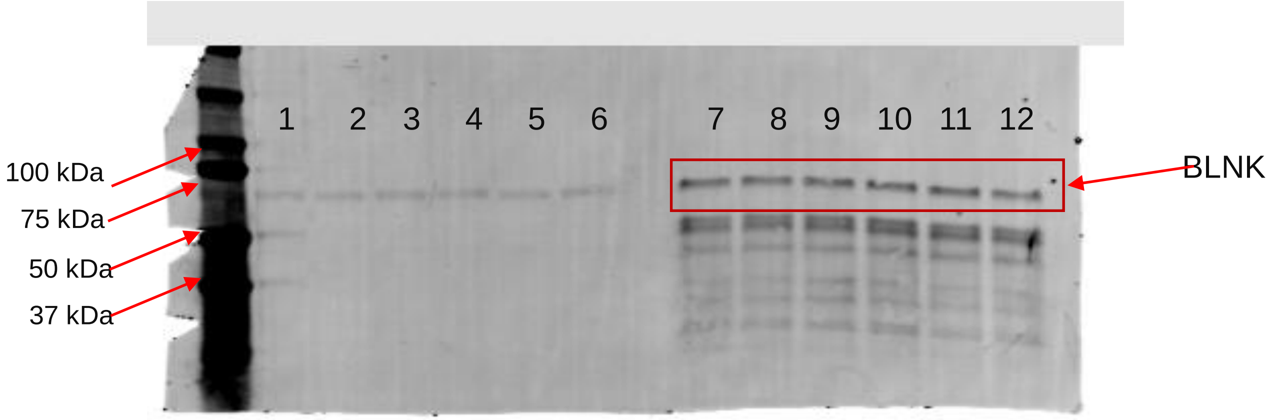

Supplementary figure 3A

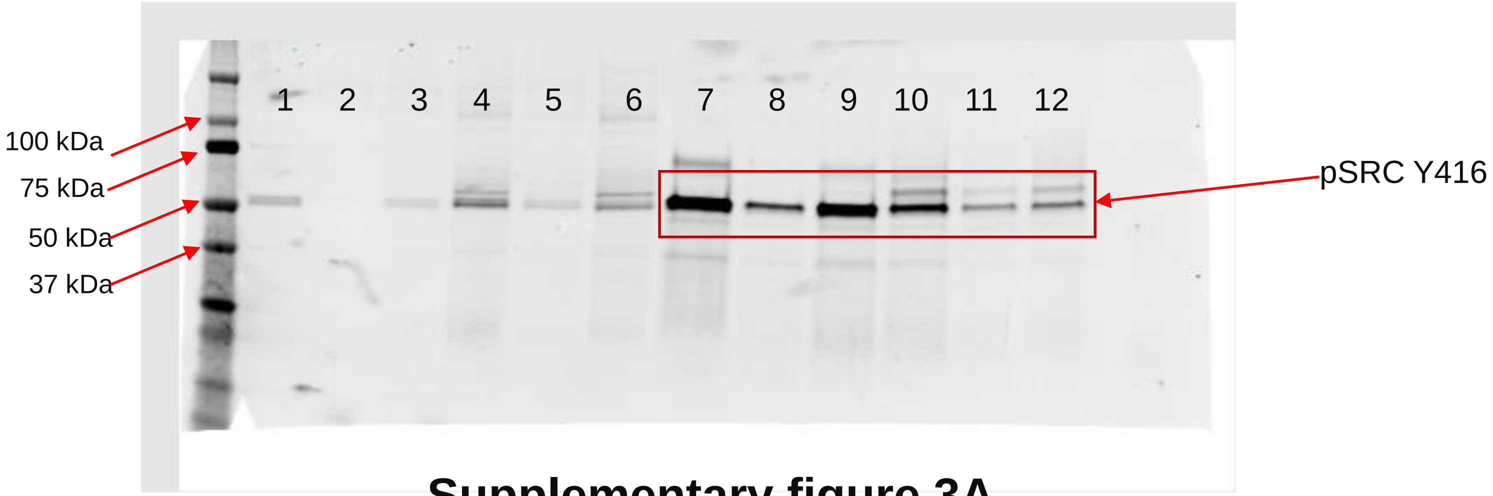

Supplementary figure 3A

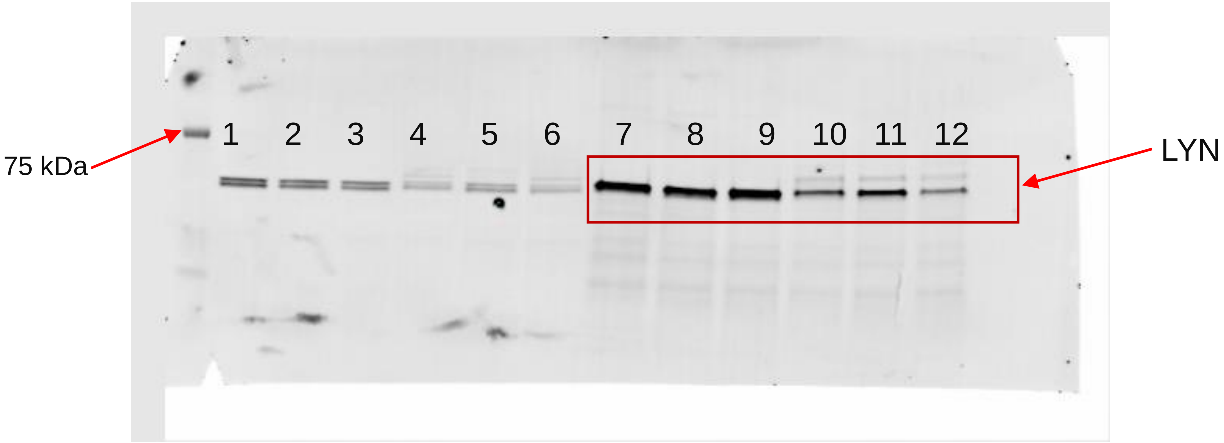

Supplementary figure 3B

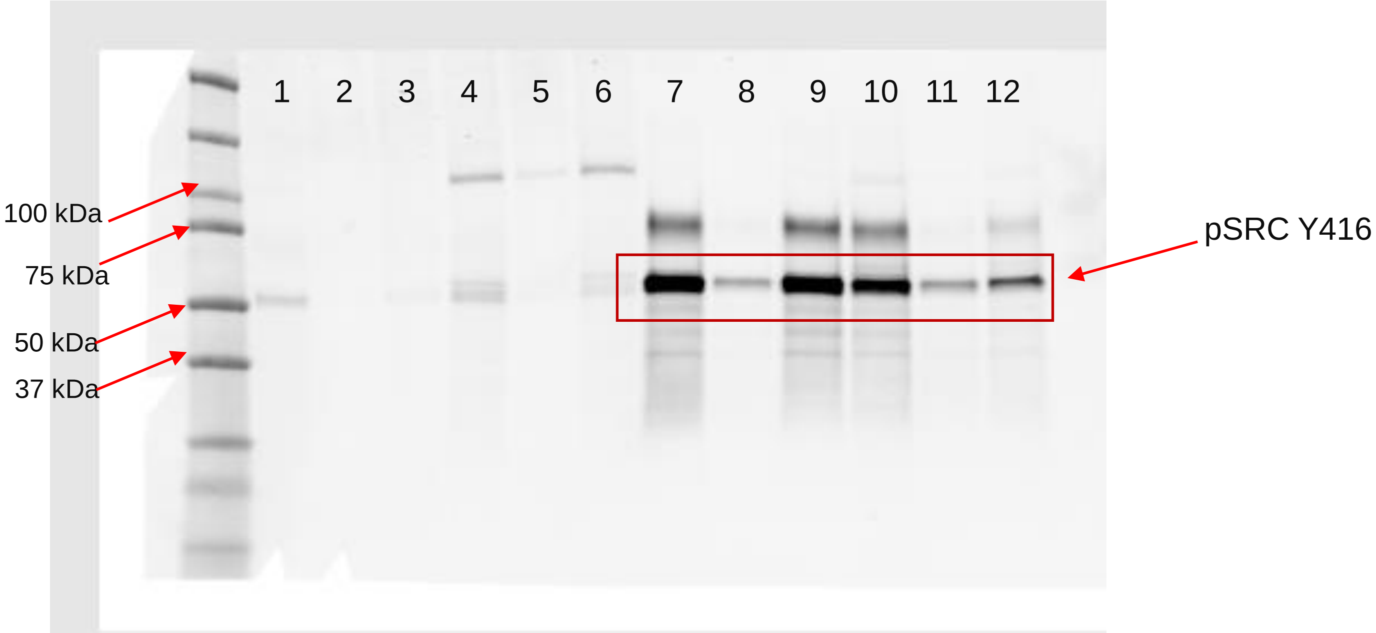

Supplementary figure 3B

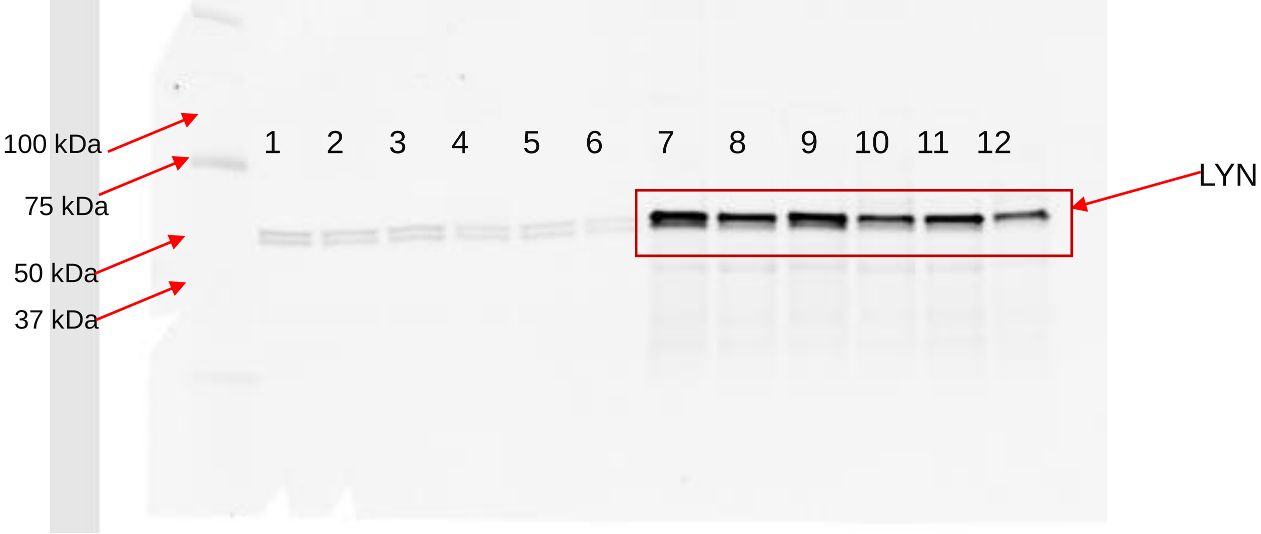

Supplement: S1 File — (PDF) [file pone.0277003.s001.pdf]
