# Supplementary figures and images for: Luxeptinib interferes with LYN-mediated activation of SYK and modulates BCR signaling in lymphoma
Source: PLoS One. 2023 Mar 8;18(3):e0277003. doi: 10.1371/journal.pone.0277003 (PMC9994718; doi:10.1371/journal.pone.0277003)

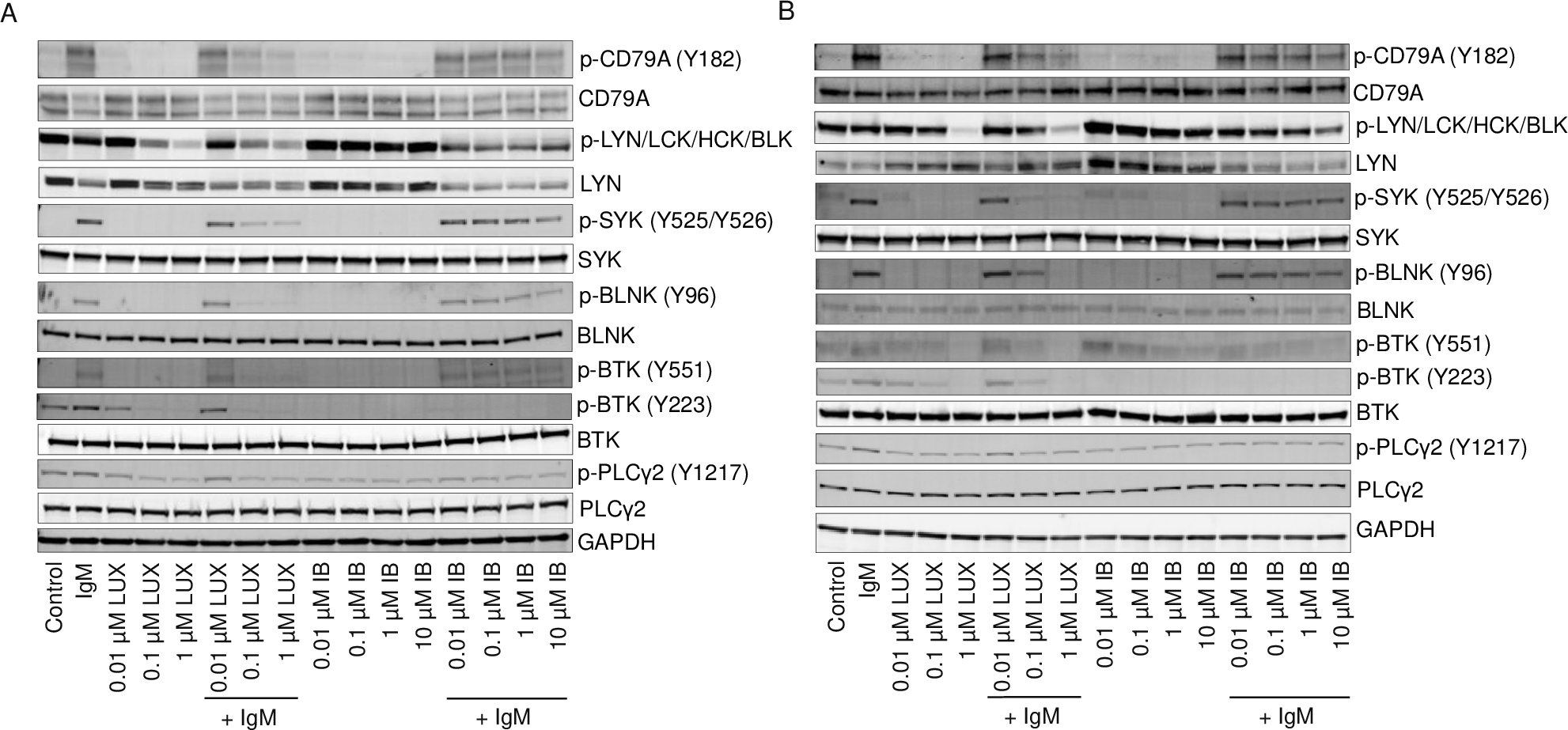

Supplement: S1 Fig — Cells were pre-treated for 2 h with either LUX or IB and then stimulated with anti-human IgM or left unstimulated. Whole cell lysates were analyzed by Western blotting for the indicated phosphoproteins in (A) JeKo-1 and (B) RL cells. Representative blots are shown. (TIF) [file pone.0277003.s002.tif]

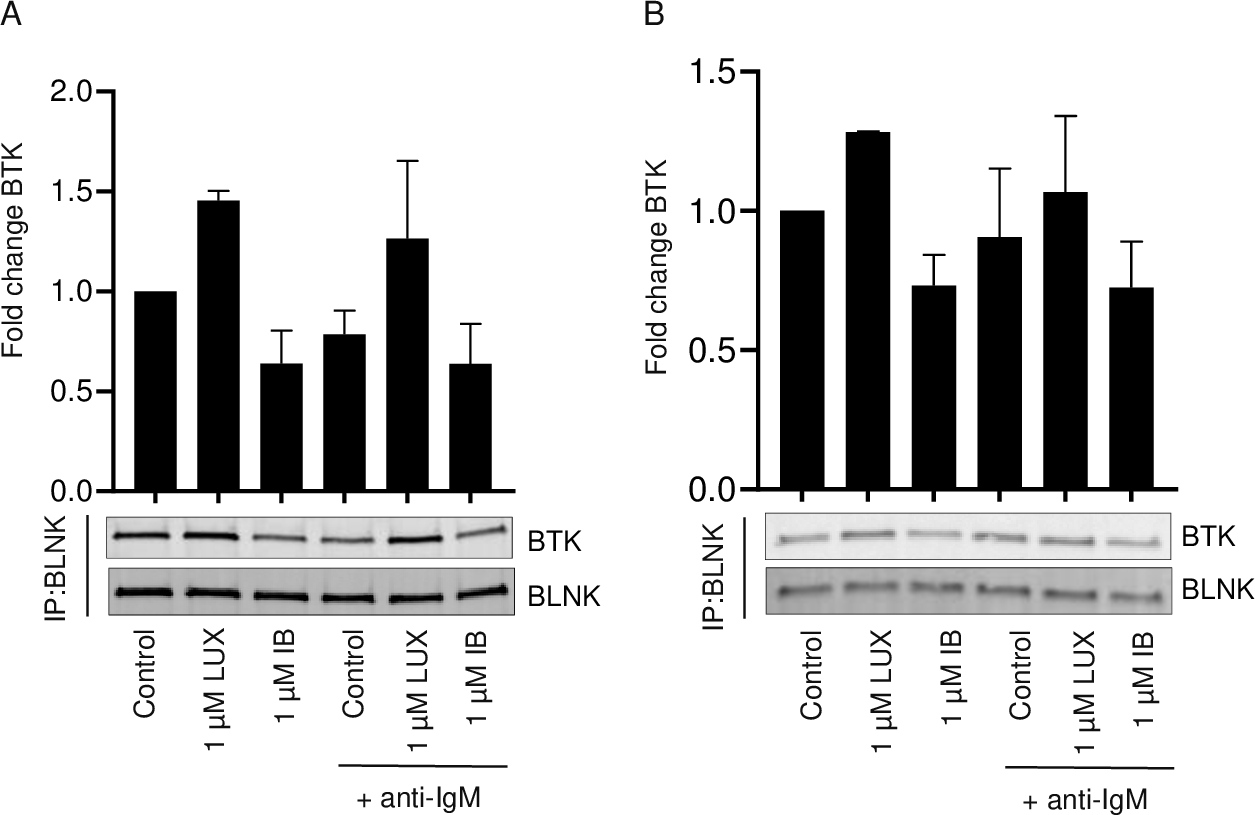

Supplement: S2 Fig — Cells were left untreated or pre-treated with 1 μM LUX or IB for 2 h followed by stimulation with anti-IgM. Representative blots showing BLNK immunoprecipitates probed for BTK and BLNK in (A) JeKo-1 and (B) RL cells sampled at 5 min. Histograms show quantification of co-precipitated protein relative to that of the protein targeted by the precipitating antibody. Bars are mean ± SEM of data from 2 independent experiments. (TIF) [file pone.0277003.s003.tif]

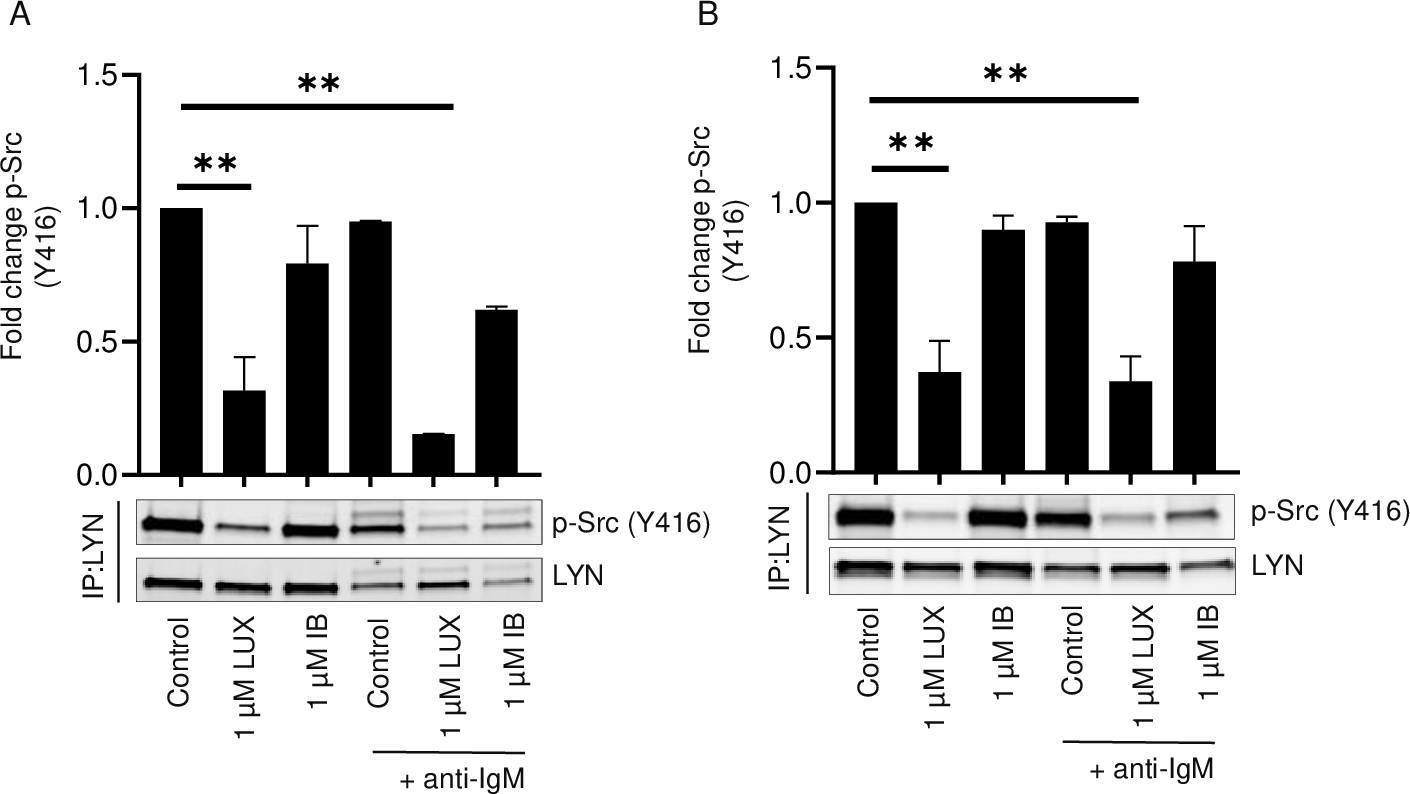

Supplement: S3 Fig — Cells were pre-treated with vehicle or 1 μM LUX or IB for 2 h followed by stimulation with anti-human IgM for 5 min. Western blot analysis of LYN immunoprecipitates using antibodies for phospho-Src (Y416) and LYN in (A) JeKo-1 and (B) RL cells. Histograms show quantification of phospho-Src (Y416) band intensity relative to that LYN. Data is mean ± SEM of data from 2 independent experiments. Representative blots are shown. **p<0.001. (TIF) [file pone.0277003.s004.tif]
