# Supplementary material for: Luxeptinib interferes with LYN-mediated activation of SYK and modulates BCR signaling in lymphoma
Source: PLoS One. 2023 Mar 8;18(3):e0277003. doi: 10.1371/journal.pone.0277003 (PMC9994718; doi:10.1371/journal.pone.0277003)
Supplement: S1 Table — Table showing IC50 values for LUX and IB in SU-DHL-6, JeKo-1 and RL cells. Data is mean ± SEM of 3-independent experiments. (DOCX) [file pone.0277003.s005.docx]

| **Supplementary Table 1. Relative sensitivity of lymphoma cell lines to LUX and IB*** | | | |
| --- | --- | --- | --- |
| **Cell Type** | **LUX IC_50_, nM** | **IB IC_50_, nM** | **Ratio** |
| SU-DHL-6 | 22 ± 0.9 | 1411 ± 38.1 | 64.1 |
| JeKo-1 | 2.1 ± 0.4 | 2633.3 ± 382.9 | 1272.1 |
| RL | 72.4 ± 21.7 | 1352.4 ± 315.5 | 18.7 |
| * Data is mean ± SEM of 3-independent experiments | | | |
